# DPHL: A DIA Pan-human Protein Mass Spectrometry Library for Robust Biomarker Discovery

**DOI:** 10.1016/j.gpb.2019.11.008

**Published:** 2020-08-12

**Authors:** Tiansheng Zhu, Yi Zhu, Yue Xuan, Huanhuan Gao, Xue Cai, Sander R. Piersma, Thang V. Pham, Tim Schelfhorst, Richard R.G.D. Haas, Irene V. Bijnsdorp, Rui Sun, Liang Yue, Guan Ruan, Qiushi Zhang, Mo Hu, Yue Zhou, Winan J. Van Houdt, Tessa Y.S. Le Large, Jacqueline Cloos, Anna Wojtuszkiewicz, Danijela Koppers-Lalic, Franziska Böttger, Chantal Scheepbouwer, Ruud H. Brakenhoff, Geert J.L.H. van Leenders, Jan N.M. Ijzermans, John W.M. Martens, Renske D.M. Steenbergen, Nicole C. Grieken, Sathiyamoorthy Selvarajan, Sangeeta Mantoo, Sze S. Lee, Serene J.Y. Yeow, Syed M.F. Alkaff, Nan Xiang, Yaoting Sun, Xiao Yi, Shaozheng Dai, Wei Liu, Tian Lu, Zhicheng Wu, Xiao Liang, Man Wang, Yingkuan Shao, Xi Zheng, Kailun Xu, Qin Yang, Yifan Meng, Cong Lu, Jiang Zhu, Jin'e Zheng, Bo Wang, Sai Lou, Yibei Dai, Chao Xu, Chenhuan Yu, Huazhong Ying, Tony K. Lim, Jianmin Wu, Xiaofei Gao, Zhongzhi Luan, Xiaodong Teng, Peng Wu, Shi'ang Huang, Zhihua Tao, Narayanan G. Iyer, Shuigeng Zhou, Wenguang Shao, Henry Lam, Ding Ma, Jiafu Ji, Oi L. Kon, Shu Zheng, Ruedi Aebersold, Connie R. Jimenez, Tiannan Guo

**Affiliations:** 1 Zhejiang Provincial Laboratory of Life Sciences and Biomedicine, Westlake University, Hangzhou 310024, China; 2 Key Laboratory of Structural Biology of Zhejiang Province, School of Life Sciences, Westlake University, Hangzhou 310024, China; 3 Institute of Basic Medical Sciences, Westlake Institute for Advanced Study, Hangzhou 310024, China; 4 School of Computer Science, Shanghai Key Laboratory of Intelligent Information Processing, Fudan University, Shanghai 200433, China; 5 Thermo Fisher Scientific (BREMEN) GmbH, Bremen 28195, Germany; 6 OncoProteomics Laboratory, Department of Medical Oncology, VU University Medical Center, VU University, Amsterdam 1011, The Netherlands; 7 Amsterdam UMC, Vrije Universiteit Amsterdam, Urology, Cancer Center Amsterdam, Amsterdam 1011, The Netherlands; 8 Thermo Fisher Scientific, Shanghai 201206, China; 9 The Netherlands Cancer Institute, Surgical Oncology, Amsterdam 1011, The Netherlands; 10 Amsterdam UMC, Vrije Universiteit Amsterdam, Surgery, Cancer Center Amsterdam, Amsterdam 1011, The Netherlands; 11 Amsterdam UMC, Vrije Universiteit Amsterdam, Pediatric Oncology/Hematology, Cancer Center Amsterdam, Amsterdam 1011, The Netherlands; 12 Amsterdam UMC, Vrije Universiteit Amsterdam, Hematology, Cancer Center Amsterdam, Amsterdam 1011, The Netherlands; 13 Amsterdam UMC, Vrije Universiteit Amsterdam, Medical Oncology, Cancer Center Amsterdam, Amsterdam 1011, The Netherlands; 14 Amsterdam UMC, Vrije Universiteit Amsterdam, Neurosurgery, Cancer Center Amsterdam, Amsterdam 1011, The Netherlands; 15 Amsterdam UMC, Vrije Universiteit Amsterdam, Pathology, Cancer Center Amsterdam, Amsterdam 1011, The Netherlands; 16 Amsterdam UMC, Vrije Universiteit Amsterdam, Otolaryngology/Head and Neck Surgery, Cancer Center Amsterdam, Amsterdam 1011, The Netherlands; 17 Erasmus MC University Medical Center, Pathology, Rotterdam 1016LV, The Netherlands; 18 Erasmus MC University Medical Center, Surgery, Rotterdam 1016LV, The Netherlands; 19 Erasmus MC University Medical Center, Medical Oncology, Rotterdam 1016LV, The Netherlands; 20 Department of Anatomical Pathology, Singapore General Hospital, Singapore 169608, Singapore; 21 Division of Medical Sciences, National Cancer Centre Singapore, Singapore 169608, Singapore; 22 School of Computer Science and Engineering, Beihang University, Beijing 100191, China; 23 MOE Key Laboratory of Carcinogenesis and Translational Research, Department of Gastrointestinal Translational Research, Peking University Cancer Hospital, Beijing 100142, China; 24 Cancer Institute (MOE Key Laboratory of Cancer Prevention and Intervention, Zhejiang Provincial Key Laboratory of Molecular Biology in Medical Sciences), The Second Affiliated Hospital, Zhejiang University School of Medicine, Hangzhou 310009, China; 25 Cancer Biology Research Center, Tongji Hospital, Tongji Medical College, Huazhong University of Science and Technology, Wuhan 430030, China; 26 Center for Stem Cell Research and Application, Union Hospital, Tongji Medical College, Huazhong University of Science and Technology, Wuhan 430030, China; 27 Department of Pathology, The First Affiliated Hospital, Zhejiang University School of Medicine, Hangzhou 310003, China; 28 Phase I Clinical Research Center, Zhejiang Provincial People's Hospital, Hangzhou 310014, China; 29 Department of Laboratory Medicine, The Second Affiliated Hospital, Zhejiang University School of Medicine, Hangzhou 310009, China; 30 College of Mathematics and Informatics, Digital Fujian Institute of Big Data Security Technology, Fujian Normal University, Fuzhou 350108, China; 31 Zhejiang Provincial Key Laboratory of Experimental Animal and Safety Evaluation, Zhejiang Academy of Medical Sciences, Hangzhou 310015, China; 32 Key Laboratory of Growth Regulation and Translational Research of Zhejiang Province, School of Life Sciences, Westlake University, Hangzhou 310024, China; 33 Department of Biology, Institute for Molecular Systems Biology, ETH Zurich, Zurich 8092, Switzerland; 34 Department of Chemical and Biological Engineering, The Hong Kong University of Science and Technology, Clear Water Bay, Kowloon, Hong Kong Special Administrative Region, China; 35 Faculty of Science, University of Zurich, Zurich 8092, Switzerland

**Keywords:** Data-independent acquisition, Parallel reaction monitoring, Spectral library, Prostate cancer, Diffuse large B cell lymphoma

## Abstract

To address the increasing need for detecting and validating protein biomarkers in clinical specimens, mass spectrometry (MS)-based targeted proteomic techniques, including the selected reaction monitoring (SRM), **parallel reaction monitoring** (PRM), and massively parallel **data-independent acquisition** (DIA), have been developed. For optimal performance, they require the fragment ion spectra of targeted peptides as prior knowledge. In this report, we describe a MS pipeline and spectral resource to support targeted proteomics studies for human tissue samples. To build the spectral resource, we integrated common open-source MS computational tools to assemble a freely accessible computational workflow based on Docker. We then applied the workflow to generate DPHL, a comprehensive DIA pan-human library, from 1096 data-dependent acquisition (DDA) MS raw files for 16 types of cancer samples. This extensive spectral resource was then applied to a proteomic study of 17 **prostate cancer** (PCa) patients. Thereafter, PRM validation was applied to a larger study of 57 PCa patients and the differential expression of three proteins in prostate tumor was validated. As a second application, the DPHL spectral resource was applied to a study consisting of plasma samples from 19 **diffuse large B cell lymphoma** (DLBCL) patients and 18 healthy control subjects. Differentially expressed proteins between DLBCL patients and healthy control subjects were detected by DIA-MS and confirmed by PRM. These data demonstrate that the DPHL supports DIA and PRM MS pipelines for robust protein biomarker discovery. DPHL is freely accessible at https://www.iprox.org/page/project.html?id=IPX0001400000.

## Introduction

The recent development of high-throughput genomic sequencing techniques, as well as methods for the global expression analysis of biomolecules, has enabled identification of a number of oncological biomarkers from clinical samples and advanced the field of cancer precision medicine [1], [2], [3], [4]. Novel diagnostic/prognostic protein marker candidates for colorectal [5], [6], breast [7], ovarian [8], and gastric [9] tumors have been identified through shotgun proteomics [10], and plasma proteomes have been reported for 1500 obese patients [11]. Sequential window acquisition of all theoretical fragment ion spectra mass spectrometry (SWATH-MS) is a massively parallel data-independent acquisition (DIA) technique that combines the multiplexing ability of shotgun proteomics with the high-accuracy data analysis of selected reaction monitoring (SRM), and can quantify proteomes using single-shot MS/MS analysis [12], [13]. The SWATH/DIA data sets are analyzed through spectral libraries using software tools like OpenSWATH [14], [15], DIA-Umpire [16], Group-DIA [17], Skyline [18], and Spectronaut [19]. Most of these tools generate comparable results [15] and require *a priori* spectral library. A pan-human spectral library (PHL) for SWATH data processing has been developed to analyze SWATH maps generated by TripleTOF MS [20] using open-source computational programs, and the error rates of peptide and protein identification in large-scale DIA analyses have been statistically controlled [21]. The development of these tools has extended the application of SWATH-MS to diverse clinical sample types including plasma [22], prostate [23], and liver [24] tissues.

Despite these advances, the implementation of DIA-MS on widely used Orbitrap instruments has currently been restricted on account of the lack of non-commercial tools to build spectral libraries. Theoretically, one could build a spectral library based on the established protocol for TripleTOF data [1]. However, in practice an optimal and robust pipeline for Orbitrap data is missing, as we have implemented in this work. Furthermore, it has been demonstrated that for Orbitrap instruments, using the library built from TripleTOF data leads to identification of much fewer proteins than using that built from Orbitrap data [25]. Moreover, there is no bioinformatics pipeline to couple DIA-MS and parallel reaction monitoring (PRM)-MS for validation, thus a comprehensive human spectral library resource for Orbitrap data is yet to be established. Parallel computing is only available for OpenSWATH software tools till now. Spectronaut has been developed to support the generation of DIA spectral libraries and analysis of DIA data sets against these libraries [19], however, it is only commercially available. To extend the application of large-scale DIA-MS on Orbitrap instruments, an open-source workflow is in great need to build a pan-human spectral library for DIA files generated for cancer biomarker discovery. Moreover, the workflow as well as spectral library are essential to validate the candidate protein biomarkers by PRM, a more recently developed technique with higher sensitivity and specificity than SWATH/ DIA, albeit with limited throughput [26].

Here, we developed an open-source computational pipeline to build spectral libraries from Orbitrap spectral data and generated DPHL, a comprehensive DIA pan-human library, from 16 different human cancer types. In addition, we have provided a Docker resource to integrate this pipeline to the data-dependent acquisition (DDA) spectral scans, which allows an easy and automatic expansion of the library by incorporating more MS data generated from ongoing studies. Finally, to validate its applicability in DIA and PRM, we applied DPHL to identify differentially expressed proteins between tumor and normal tissues in a prostate cancer (PCa) cohort and a diffuse large B cell lymphoma (DLBCL) cohort, respectively.

## Results and discussion

### Build DPHL using 1096 shotgun proteomics data files

**Figure 1:**
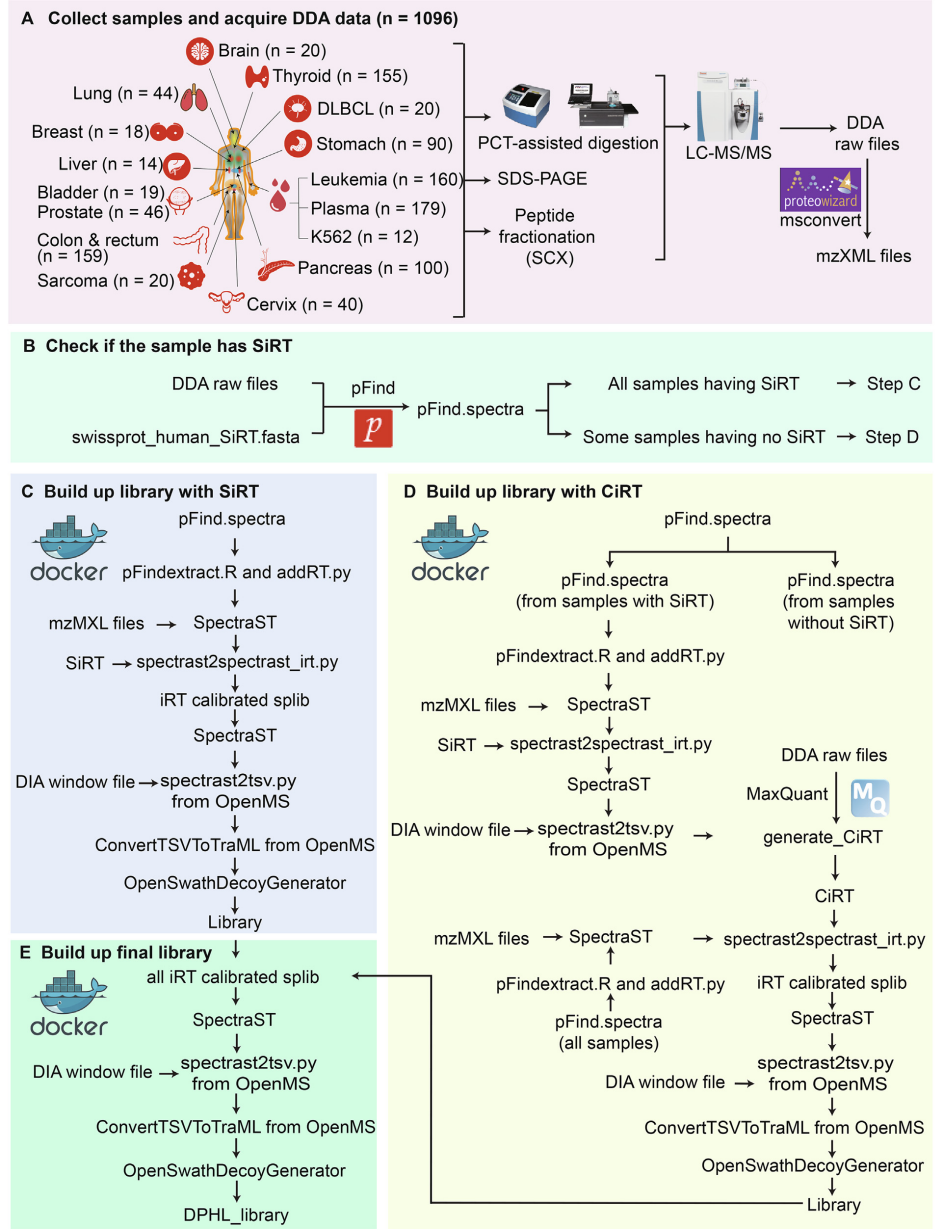
**Workflow for building DPHL** **A.** Schematic representation of DDA shotgun proteomics data acquisition. Numbers in parentheses indicate the number of DDA files per tissue type. **B.** Protein identification and iRT detection from DDA raw files using pFind. **C.** SiRT detection and calibration. **D.** CiRT detection and calibration. **E.** Generation of DPHL. Details of the commands are presented in File S18. DDA, data-dependent acquisition; DIA, data-independent acquisition; iRT, indexed retention time; PCT, pressure cycling technology; SCX, strong cation-exchange; SiRT, synthetic iRT; CiRT, common internal iRT; DPHL, DIA pan-human library.

To build a DIA spectral library for Orbitrap data that can also be used for PRM assay generation, we collected shotgun proteomics data from two laboratories. These two laboratories, the Guo lab from China and the Jimenez lab from the Netherlands, used Q Exactive HF mass spectrometers and consistent experimental conditions (see Materials and Methods section). A total of 1096 raw MS data files were collected from a range of samples that included tissue biopsies from PCa, cervical cancer (CC), colorectal cancer (CRC), hepatocellular carcinoma (HCC), gastric cancer (GC), lung adenocarcinoma (LADC), squamous cell lung carcinoma (SCLC), thyroid diseases, glioblastoma (GBM), triple-negative breast cancer (TNBC), sarcoma, and DLBCL (Figure 1A). In addition, blood plasma samples from acute myelocytic leukemia (AML), acute lymphoblastic leukemia (ALL), chronic myelogenous leukemia (CML), multiple myeloma (MM), myelodysplastic syndrome (MDS), and DLBCL patients, as well as the human CML cell line K562 were also analyzed, with the resulting data included in the library too. The sample types and their DDA files are summarized in Figure 1A and Table S1A. Comparison of DDA files acquired from the Guo lab and the Jimenez lab demonstrated a high degree of consistency (File S1).

### Establish an open-source Docker-based computational pipeline for building DIA/PRM spectral library

The conventional OpenMS and OpenSWATH pipeline [14] requires sophisticated installation, which relies on multiple existing software packages. A Docker image can largely facilitate the installation process. We developed an open-source Docker image with all the pre-installed pipelines and its dependent packages to democratize the generation of DIA/PRM spectral libraries. The workflow of this computational pipeline is shown in Figure 1. Briefly, the DDA files were firstly centroided and converted to mzXML using MSconvert from ProteoWizard [27] (Figure 1A), and pFind [28] was used to identify the relevant peptides and proteins in the protein database (Figure 1B). The shotgun data from each tissue type were processed separately. We wrote two scripts, namely pFindextract.R and addRT.py (Figure 1C and D), to extract the retention time (RT), peptide sequence, charge state, protein name, and identification score for each peptide precursor. SpectraST version 5.0 [29] was used to generate consensus spectra of peptides for each tissue type to build the library; spectrast2spectrast_irt.py [30] was used to calibrate RT; and spectrast2tsv.py [14] was used to select the top six fragments for each peptide precursor (Figure 1C and D). Decoy assays were generated using OpenSwathDecoyGenerator from OpenSWATH software [14].

For both library building and SWATH/DIA analysis, the synthetic indexed RT (SiRT) peptides [31] were spiked in the peptide samples for RT calibration (Figure 1C), and these samples were subjected to SWATH library building workflow [1]. For some samples without SiRT spike-in, we developed software tools to identify the conserved high-abundance peptides with common indexed RT (CiRT) (Figure 1D) [30]. The peptides of each tissue type had to fulfill the following criteria to be considered as CiRT peptides: (1) proteotypic, (2) amino acid sequences with no modification, (3) signal intensities above the 3rd quartile of all quantified peptide precursors, (4) charge +2 or +3, and (5) uniformly distributed RT across the entire liquid chromatography (LC) gradient. Following these criteria, we implemented codes dividing the LC gradient window into 20 bins and selected one peptide for each bin. Thereby we selected 20 CiRT peptides for each tissue type. The detailed information of CiRT peptides in different tissue types is shown in Table S2A and CiRT peptides of each tissue type are shown in Table S2B. The transitions markup language (TraML) format of the CiRT peptides is provided in File S2–S17. The CiRT peptides can either be used unified with exogenous SiRT standards or as an alternative RT standard in the respective samples. We expect these CiRT peptides to be of wide use in future DIA experiments for clinical tissue samples.

Since the current version of the pFind software does not support the quantification of identified peptides, CiRT peptides were selected from a representative DDA dataset that was analyzed by MaxQuant (version 1.6.2) [32]. We then wrote the generate_CiRT.R script to analyze the peptides.txt files from the MaxQuant search results, and generated the tissue-specific CiRT peptides (Figure 1D). The latter was used to replace SiRT peptides in the command spectrast2spectrast_irt.py [30]. For RT calibration, we used the spectrast2spectrast_irt.py converter script on the SiRT or CiRT peptides. Then, SpectraST was used to build a consensus library, and spectrast2tsv.py and OpenSwathDecoyGenerator [14] were applied to filter low quality assays and append decoy assays into the library (Figure 1E). The computational pipeline is illustrated and explained in more detail in File S18 and File S19.

### DPHL is to date the most comprehensive DIA/PRM library for human specimens

**Figure 2:**
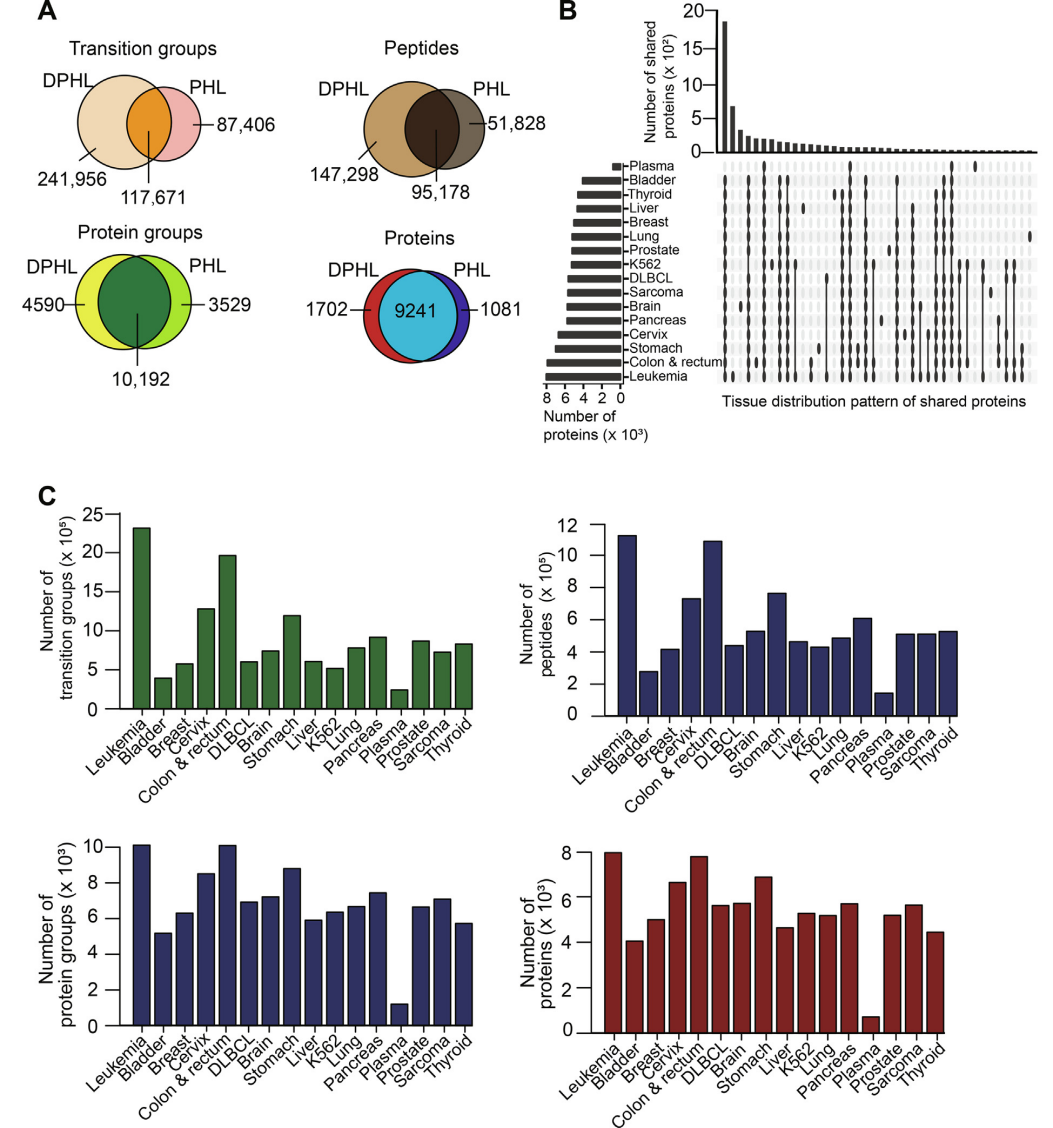
**Comparison of DPHL and PHL** **A.** Venn diagram showing the comparison of transition groups (*i.e.*, peptide precursors), peptides, protein groups, and proteins in DPHL and PHL. **B.** Visualization of tissue intersections using R package UpSet. **C.** Bar plots displaying the number of transition groups, peptides, protein groups, proteins in DPHL library for each sample type. PHL, pan-human spectral library.

We first characterized the content of the newly-generated DPHL library in terms of the peptide and protein identification, and compared it to the PHL library for SWATH [20]. The DPHL library includes 359,627 transition groups (referred as peptide precursors thereafter), 242,476 peptides, 14,782 protein groups, and 10,943 proteotypic Swiss-Prot proteins (referred as proteins thereafter for short). DPHL and PHL share 9241 proteins, which represent 84.4% content of DPHL and 89.5% content of PHL (Figure 2A). The DPHL library includes more peptide precursors, peptide, protein groups, and proteins compared to the PHL library for SWATH (Figure 2A). Proteins in DPHL are of higher sequence coverage (Figure S1), enabling better measurement of specific domains of proteins.

We then counted the number of peptide precursors, peptides, and protein groups for each of the 16 sample types (Figure 2B) and found that the solid tissues, but not the plasma samples, shared a large number of proteins. The leukemia samples had the highest number of peptides and proteins due to the higher number of DDA files (*n* = 160) available (Figure 2C). The plasma samples had, as expected, the lowest number of peptides and proteins due to the dominance of high abundance proteins (Figure 2C). Cumulative plots of peptides and proteins of the 16 types of cancer samples (tissue, plasma, and cell line) are shown in Figure S2A and B. There was a significant increase in the number of transition groups when DDA data was added from different tissue types (Figure S2A), while the increase in the number of proteins was relatively low (Figure S2B). We further investigated the increase in the number of peptide precursors and proteins in two well-sampled tissue types and found that the current DPHL library is not yet complete, probably due to the presence of semi-tryptic peptides, missed cleavages, and biological heterogeneity (Figure S2C and D), waiting for future expansion with more spectral data.

Next, we analyzed the biological content of the DPHL library. To investigate the biological coverage of DPHL, we did Gene Ontology (GO) enrichment analysis using R package clusterProfiler. It is shown that DPHL covers proteins with diverse molecular functions (Figure S3).

The kinases were next characterized using KinMap [33], an online tool that links the biochemical, structural, and disease association data of individual kinases to the human kinome tree. A total of 340 kinases (63.2% out of 538 known protein kinases) identified in DPHL were plotted in the KinMap tree. As shown in Figure S4, DPHL covers all the major branches of the kinome tree. More characteristics of the kinases in DPHL are shown in Figure S5. Given that transcription factors (TFs) are extremely important to disease genesis, development, and disease progression, we matched DPHL to the 1639 TFs from the Human Transcription Factors database [34]. We found that DPHL covers 33.0% of the known TFs (Figure S6).

### DPHL assists stratification of PCa tissue samples and discovery of potential protein biomarkers

**Figure 3:**
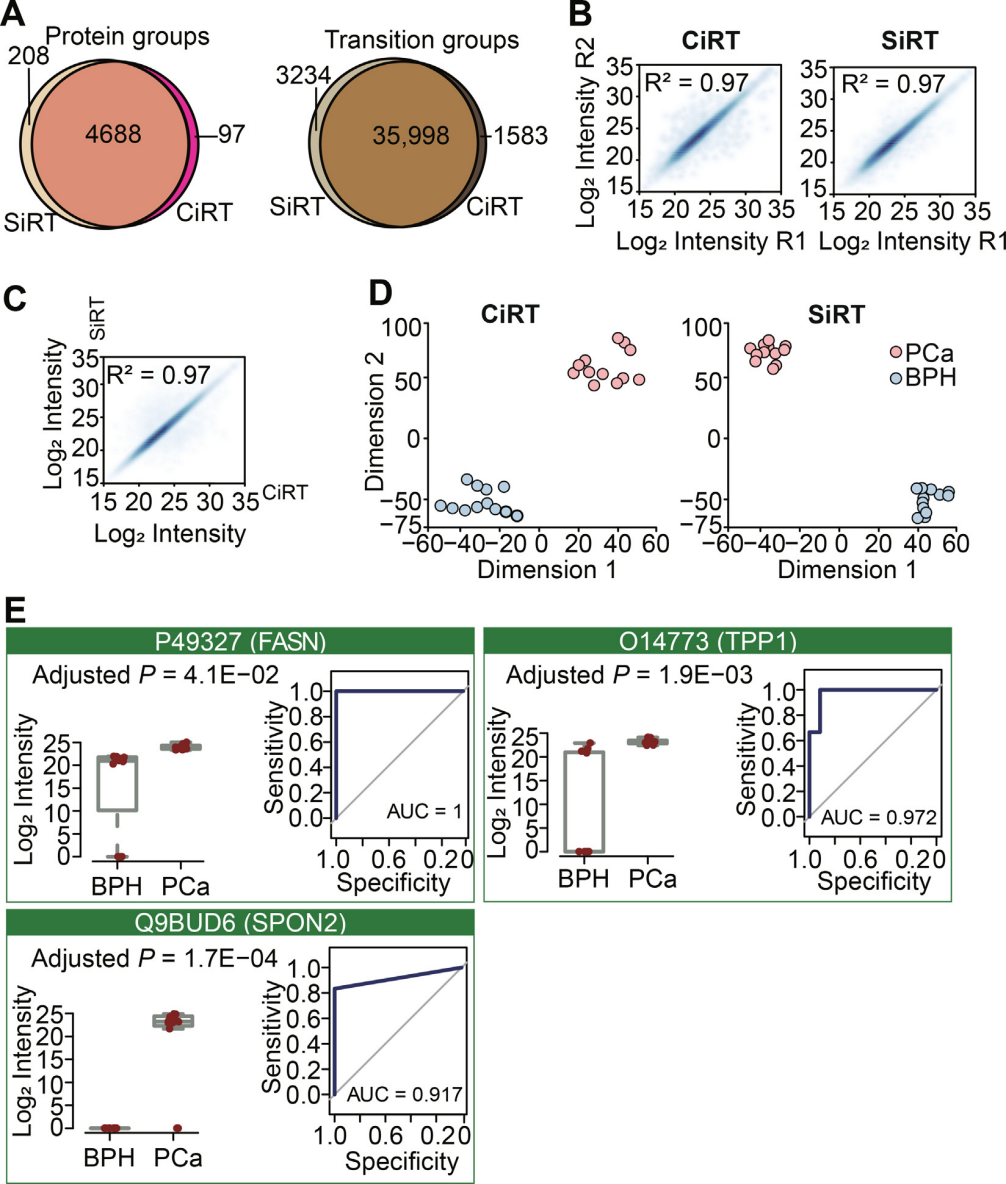
**PCa proteome using 60-min gradient DIA** **A.** Number of protein groups and peptide precursors identified using SiRT and CiRT. **B.** Technical reproducibility of proteome matrix using CiRT and SiRT. **C.** Comparison of protein quantification based on MS intensity using the SiRT and CiRT methods. **D.** 2D plane t-SNE plot of disease classes, color coded by sample type using CiRT and SiRT. **E.** Boxplots showing the expression (MS intensity) of the significantly dysregulated proteins; *P* values adjusted with Benjamini & Hochberg are shown under each protein name. ROC curves of the proteins were also shown. R1, technical replicate 1; R2, technical replicate 2; PCa, prostate cancer; BPH, benign prostate hyperplasia; t-SNE, t-distributed stochastic neighbor embedding; FASN, fatty acid synthetase, UniProtKB: P49327; TPP1, tripeptidyl-peptidase 1, UniProtKB: O14773; SPON2, spondin-2, UniProtKB: Q9BUD6.

Next, we applied DPHL to analyze representative clinical sample cohorts. First, we procured prostate tissue samples from 17 patients consisting of 8 PCa cases and 9 benign prostate hyperplasia (BPH) cases (Table S3A and B) for analysis on Q-Exactive HF MS operated in DIA mode. The peptides were separated on a 60-min LC gradient. Two additional technical replicates were randomly selected for each patient group. As a result, 24 DIA files were acquired; 4785 protein groups and 3723 proteins were identified from 37,581 peptide precursors that were searched against DPHL using the CiRT strategy. CiRT and SiRT strategy shared most of protein groups and peptide precursors (Figure 3A, Table S3C). Figure 3B shows that proteins were identified at a high degree of reproducibility across the samples tested. The SiRT (Table S3D) and CiRT strategies achieved comparable performance (Figure 3C). T-distributed stochastic neighbor embedding (t-SNE) [35] plots show that PCa and BPH samples were clearly distinguished by the data analyzed by both the CiRT and SiRT strategies (Figure 3D).

Of the 3723 identified proteins, 1555 (1451 up-regulated and 104 down-regulated) proteins showed significantly differential abundance [Benjamini–Hochberg (BH) adjusted *P* < 0.05 and intensity absolute fold change |FC| ≥ 2] using the CiRT strategy, compared to 2109 (1954 up-regulated and 155 down-regulated) proteins identified using the SiRT strategy (Table S3E and F). The two regulated proteomes shared 1359 proteins in common. Then, random forest (RF, using R package ‘randomForest’) analysis was applied to the 1359 common proteins to select prioritized proteins to distinguish benign and malignant tumor samples in this PCa patient cohort, leading to a shortlist of 400 proteins. We further analyzed the 400 proteins using Metascape [36] based on pathway and protein interaction, resulting in a further refined shortlist of 86 proteins (Table S4A–C). Among these 86 proteins, we focused on three biomarker candidates, *i.e.*, fatty acid synthase (FASN; UniProtKB: P49327), tripeptidyl-peptidase 1 (TPP1; UniProtKB: O14773), and spondin-2 (SPON2; UniProtKB: Q9BUD6), based on their functional annotation. FASN, TPP1, and SPON2 are all significantly upregulated in this tumorous samples (Figure 3E). FASN overexpression has been reported to be associated with poor prognosis in PCa [37]. TPP1 regulates single-stranded telomere DNA binding and telomere recruitment, thus maintaining telomere stability [38], [39], [40]. Since genomic instability drives PCa progression from androgen-dependence to castration resistance [41], TPP1 is a promising biomarker [42]. SPON2, a cell adhesion protein that plays a role in tumor progression and metastasis, has been reported as a serum biomarker [43], [44], [45]. The receiver operating characteristic (ROC) curves of these three proteins were shown in Figure 3E, and the high area under curve (AUC) values suggest these proteins as potential markers for PCa.

### DPHL assists stratification of DLBCL plasma samples and discovery of potential protein biomarkers

**Figure 4:**
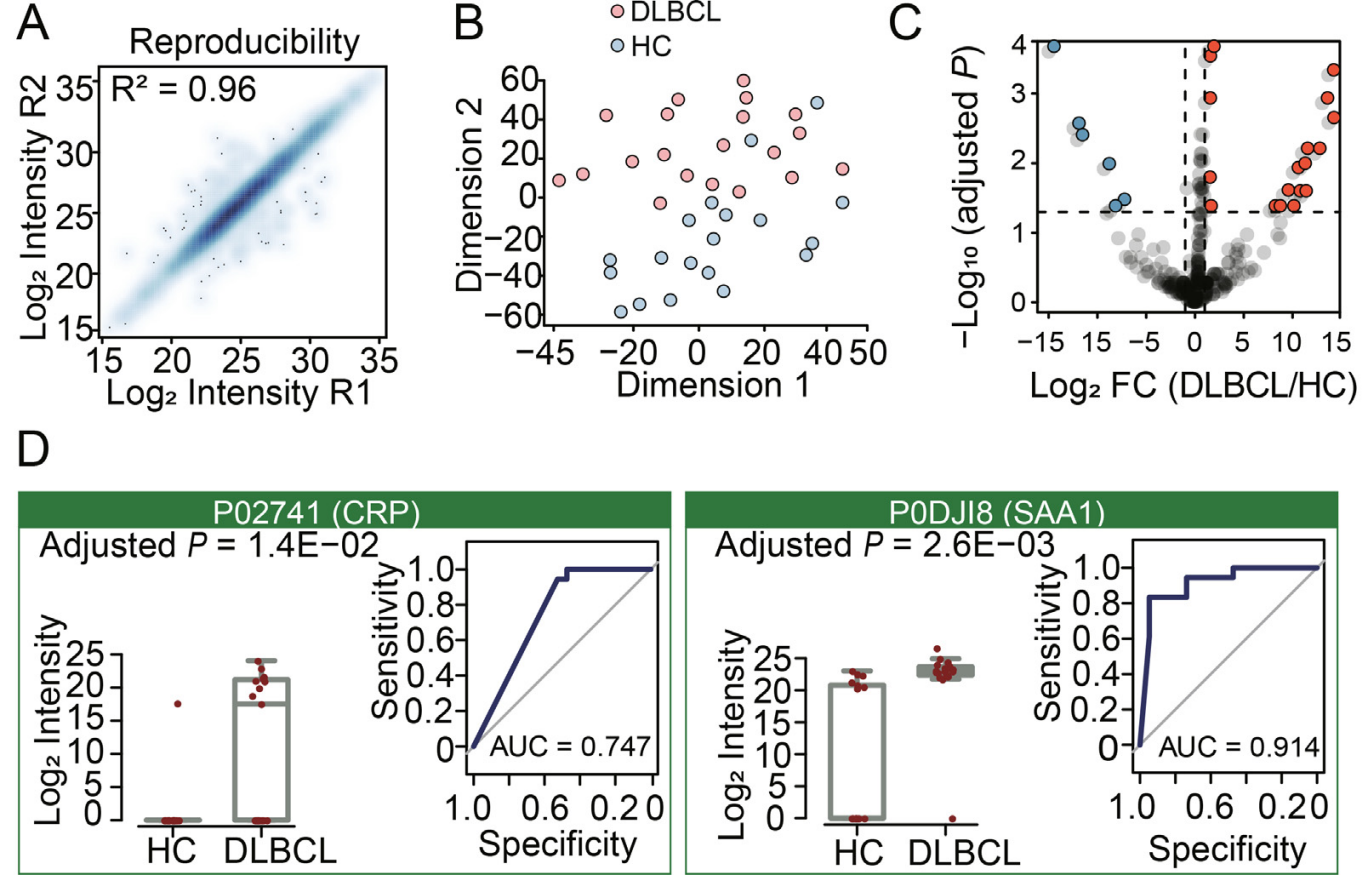
**DIA analysis of plasma samples from DLBCL patients and HC subjects** **A.** Technical reproducibility for protein quantification of four plasma samples from two DLBCL patients and two healthy control subjects. **B.** 2D plane t-SNE plot showing that proteomes are separated. **C.** Volcano plot showing significantly down-regulated (blue) and up-regulated (red) proteins in 37 plasma samples (19 samples from DLBCL patients and 18 samples from HC subjects). **D.** The relative protein expression as calculated by MS intensity for CRP and SAA1. *P* values adjusted with Benjamini & Hochberg are shown under each protein name Left: Boxplot and ROC curve of CRP. Right: Boxplot and ROC curve of SAA1. DLBCL, diffuse large B cell lymphoma; HC, healthy control; CRP, C-reactive protein, UniProtKB: P02741; SAA1, serum amyloid A1, UniProtKB: P0DJI8.

Plasma has been widely used in clinical diagnosis for its convenient access. Here we applied DIA-MS and DPHL to analyze the plasma samples from DLBCL patients. The plasma samples were procured from 19 DLBCL patients and 18 healthy control subjects (Table S5A–C). Each unfractionated and un-depleted plasma sample was trypsinized and the resulting peptides were separated on a 20-min LC gradient and measured by DIA-MS on a Q-Exactive HF instrument. A total of 7333 peptide precursors were identified by searching the data against the DPHL plasma subset library using the CiRT strategy with high technical reproducibility (*R*^2^ = 0.96, Figure 4A). We identified 507 protein groups and 304 proteins. More detailed information for each sample is shown in Figure S7. The DLBCL samples were clearly distinguished from the healthy control samples by t-SNE analysis of the quantified proteome (Figure 4B), indicating that our workflow can distinguish DLBCL patients from healthy control subjects based on their plasma proteomes.

After comparing the plasma proteomes of DLBCL with healthy control (or normal) samples using *t*-test using the same criteria as in the prostate cohort, we identified 24 differentially regulated proteins (Figure 4C, Table S5D). Two protein candidates that were closely associated with DLBCL were chosen for further investigation in this study, including C-reactive protein (CRP; UniProtKB: P02741) and serum amyloid A1 (SAA1; UniProtKB: P0DJI8). CRP is an indicator of the inflammatory response and has prognostic value in various solid tumors, including DLBCL [46]. The hyaluronic acid receptor SAA1 has been previously identified as a prognostic biomarker for DLBCL [47], [48]. The boxplots and ROC curves of these proteins are shown in Figure 4D. Our data support that DPHL is an effective resource for DIA-based discovery of potential prognostic biomarkers of DLBCL.

### DPHL assists protein validation using PRM

We then validated the candidate biomarkers using PRM, a highly specific and sensitive mass spectrometric method that can precisely and systematically quantify peptides in highly complex samples. The DPHL spectra were used to develop PRM assays using Skyline [18].

#### Validation in prostate samples

**Figure 5:**
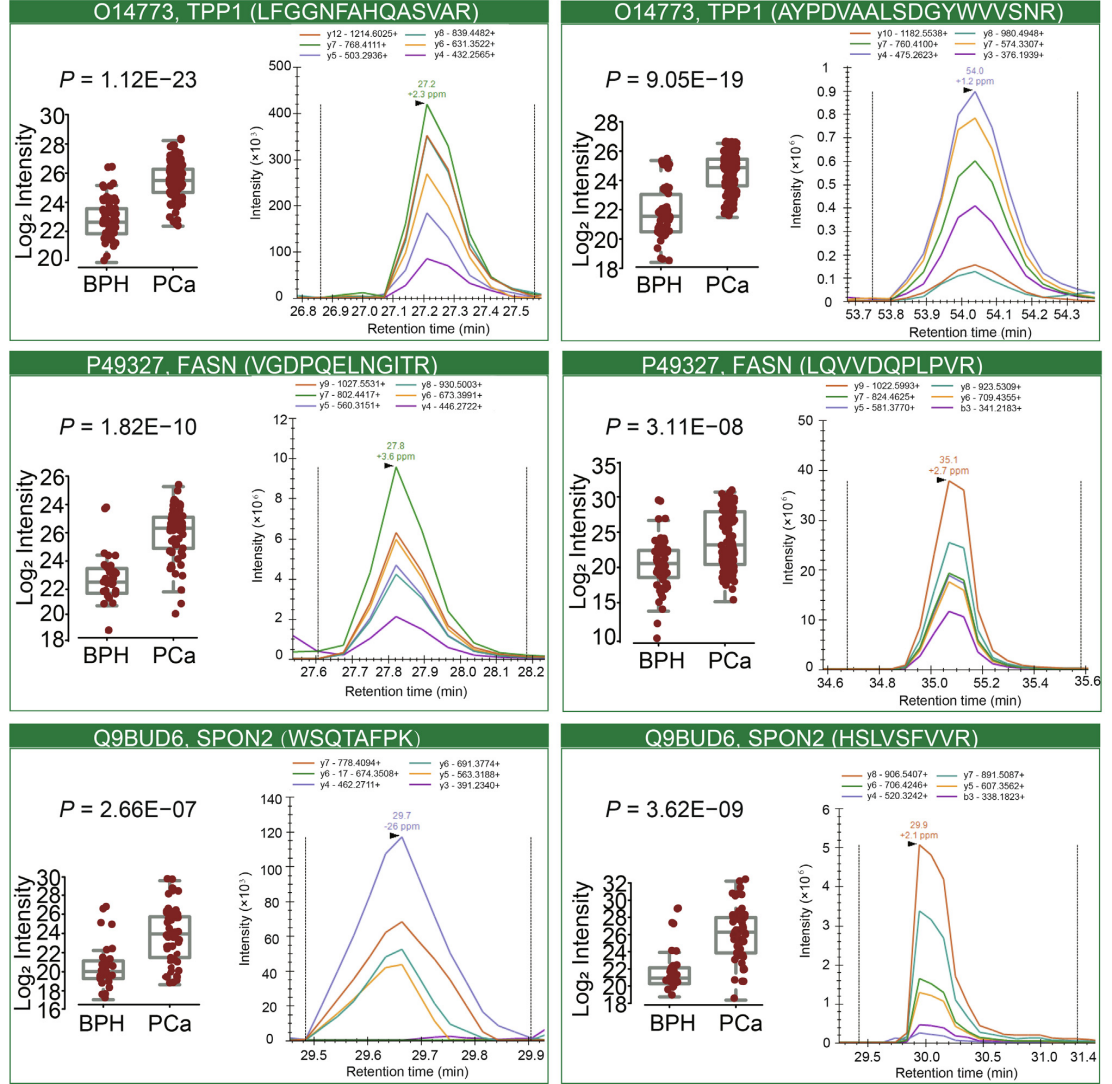
**PRM validation of TPP1, FASN, and SPON2 across 73 peptide samples from 53 PCa patients** Two best flying peptides were selected for each protein. For each peptide, boxplot shows the relative abundance of the peptide across 73 PRM runs as calculated from MS intensity (on the left), and XIC demonstrates a representative peak group of the peptide (on the right). *P* values are computed using Student’s *t* test. PRM, parallel reaction monitoring; XIC, extracted ion chromatogram.

To validate the DIA results of the prostrate samples, we included another independent PCa cohort including 73 samples from 57 patients (Table S6A–C). The two best flying peptides were selected for each protein to measure the abundance of FASN, TPP1, and SPON2 (Figure 5). As shown in Figure 3E and Figure 5, PRM data exhibited good consistency with the DIA results. As a representative example, the peak areas of protein TPP1 (UniProtKB: O14773) across all samples are shown in Figure S8.

#### Validation in plasma samples

The putative DLBCL biomarkers CRP and SAA1 that were identified from the DIA dataset were selected for PRM validation, using the same cohort as DIA (Table S7A–C). Skyline was used to visualize characteristic peptides for CRP and SAA1. One of the best flying peptides were selected for CRP and SAA1 to measure the abundance of each protein (Figure S9). The peak groups of the fragment ions were manually curated. As shown in Figure S9, amounts of both proteins are highly upregulated in DLBCL patients compared to healthy control subjects, confirming the results obtained by DIA (Figure 4D). As an example, the peak areas of peptide ESDTSYVSLK (m/z of 564.77) of CRP across all samples are shown in Figure S10.

## Conclusion

In this study, we have developed an open-source platform consisting of a computational pipeline to generate spectral libraries for DIA and PRM analyses on Orbitrap instruments. We also reported a reference spectral library that can be used to identify and validate protein biomarkers in clinical samples using DIA-MS. With over 370,000 peptide precursors and more than 10,000 proteins identified, DPHL is the most comprehensive human DIA library built to date and allows convenient partitioning into tissue-specific and disease-specific sub-libraries. Additionally, DPHL is specifically designed for protein measurement of clinical samples including tissues and plasma, while PHL is mainly for cell lines and synthetic peptides. With the established pipeline and DPHL library, we are able to analyze the proteomes of 20 human tissue samples or 40 plasma samples per MS instrument per day. We will continue to generate additional DDA files from more types of human tumors with the ambition of incorporating internal and external data to create a comprehensive resource, reflecting tumor heterogeneity that enables biomarker discovery as a mission of the Human Cancer Proteome Project (Cancer-HPP) of the Human Proteome Organization [49]. By appending these results to DPHL, we can increase the human proteome coverage. Users could add their own DDA files to DPHL to extend the library, following the instructions in File S18 (Part 4). The pipeline is robust to different experiment conditions. DPHL itself is composed of DDA files produced by two different labs with slightly different LC columns and gradients. The data consistency of DDA files acquired from the Guo lab and the Jimenez lab to constitute DPHL was high (File S1). DPHL is not only applicable to open-source SWATH/DIA analysis tools like OpenSWATH, but also to other tools including Spectronaut and Skyline, among others.

## Materials and methods

All chemicals were purchased from Sigma unless otherwise stated. All MS grade reagents for MS analyses were obtained from Thermo Fisher Scientific (Waltham, MA).

### Clinical samples

Formalin-fixed paraffin-embedded (FFPE), fresh or fresh frozen (FF) tissue biopsies from PCa, CC, CRC, HCC, GC, LADC, SCLC, thyroid diseases, GBM, sarcoma, and DLBCL were analyzed in this study. Human plasma samples from a range of types of leukemia, lymphoma, plasma cell disorders, anemia, and DLBCL were also included. The human chronic myelocytic leukemia (CML) cell line, K562, was present in the dataset. The details about the samples are described in Table S1A.

### Cancer tissue cohorts from China

PCa FFPE samples were acquired from the Second Affiliated Hospital of Zhejiang University School of Medicine, China. The first cohort including 3 PCa patients and 3 BPH patients was used for DPHL library building. The second cohort containing 8 PCa patients and 9 BPH patients was selected for DIA-MS proteotyping. For each patient, four tissue biopsies (punch 1 × 1 × 5 mm^3^) from the same region were procured for the subsequent FFPE pressure cycling technology (PCT)-SWATH/DIA workflow for targeted quantitative proteomics profiling [50]. Besides the second cohort, a third cohort including 57 patients (10 BPH and 47 PCa) was also included for PRM validation. PRM and DIA analyses were performed in technical duplicate. Information about samples of patient used for DIA and PRM measurements are described in Tables S3 and S6.

The CRC cohort was acquired from histologically confirmed tumors at the First Affiliated Hospital of Zhejiang University School of Medicine and the Second Affiliated Hospital of Zhejiang University School of Medicine. Among the 15 donors, eight patients were diagnosed with colorectal adenocarcinoma, one patient with mucinous adenocarcinoma, three patients with adenoma, two patients with polyps, and one with benign tissue at the edge of colorectal tumors. The CRC cohort of 15 donors consisted of FFPE and FF tissue samples. These samples (1.5 × 1.5 × 5 mm^3^ in size) were punched from pathologically confirmed tissue areas using Manual Tissue Arrayer MTA-1 (Beecher Instruments, Sun Prairie, WI). FF tissue samples were snap frozen and stored in liquid nitrogen immediately after surgery and were transported to the proteomics lab within 24 h.

The HCC cohort and LADC cohort were collected from Union hospital, Tongji Medical College, Huazhong University of Science and Technology. 66 tissue samples (benign and tumor) from 33 HCC patients were collected within 1 h after hepatectomy, then snap frozen and stored at −80 °C. Similarly, 16 tissue samples (matched benign and tumor pairs) from eight LADC patients were collected within 1 h after pneumonectomy, then snap frozen and stored at −80 °C.

The CC cohort was collected from Tongji Hospital, Tongji Medical College, Huazhong University of Science and Technology. 13 FFPE cancerous and benign tissues were obtained from patients with operable CC.

### Cancer plasma cohorts from China

Pooled plasma for building the plasma library was created by mixing plasma (10 μl for each patients) from 20 patients from Union Hospital, Tongji Medical College, Huazhong University of Science and Technology. Each of the 20 patients had one of the following: AML, ALL, CML, MM, MDS, and DLBCL. The validation cohort consisted of two groups: 18 clinically healthy control subjects from the Second Affiliated Hospital, Zhejiang University School of Medicine, and 19 patients diagnosed with DLBCL from Union Hospital, Tongji Medical College, Huazhong University of Science and Technology.

### Cancer tissue cohorts from the Netherlands

The GBM, DLBCL, AML, ALL, CC, pancreatic, and GC cohorts were collected at Amsterdam UMC/VU Medical Center, Amsterdam. mirVana acetone precipitations of 19 GBM cancer tissues were pooled according to epidermal growth factor receptor (EGFR) status (10 samples with wild-type EGFR and 9 samples with mutant (vIII) EGFR). Similarly, mirVana acetone precipitations of samples from 27 DLBCL patients were pooled according to origin (12 samples of neck origin and 17 of non-neck origin). For AML, two pools of two patient samples each were prepared. For ALL, 14 individual primary ALL cell samples were used, including nine glucocorticoid-resistant and five glucocorticoid-sensitive samples. CC tissue lysates of 16 patients were prepared and pooled. For pancreatic cancer, individual tissue lysates of 20 patients were used. For GC, tissues in the form of FFPE material of 10 patients were pooled according to tumor percentage (7 with > 50% and 3 with ≤ 50% tumor content).

The lung cancer cohort was acquired from Amsterdam UMC/VU Medical Center, Amsterdam and Antoni van Leeuwenhoek Hospital/Netherlands Cancer Institute, Amsterdam. Tumor resection samples in the form of FFPE material were collected from 10 LADC, 10 SCLC, and three large cell lung carcinoma (LCLC) patients and pooled per subtype.

The soft tissue sarcoma cohort was acquired from Antoni van Leeuwenhoek Hospital/Netherlands Cancer Institute, Amsterdam. Seven sarcoma and nine sarcoma metastasis tissues were pooled separately.

PCa and bladder cancer cohorts were acquired from Amsterdam UMC/VU Medical Center, Amsterdam and Erasmus University Medical Center, Rotterdam. In total 18 PCa tissues and nine healthy control tissues in the form of FFPE material were pooled separately. In addition, 22 FF PCa tissues were combined into two pools of 11 samples each. Additionally, 10 bladder cancer tissues in the form of FFPE material were combined into two pools of five samples each.

The CRC and TNBC cohorts were collected at Erasmus University Medical Center, Rotterdam. For CRC, two pools were made per consensus molecular subtypes (CMS1, 2, 3, and 4), with each pool containing tissue lysates of five patients. For TNBC, two pools of 23 and 24 patient tissues each were used.

### Thyroid cancer cohort from Singapore

The thyroid tissue cohort was kindly provided by the National Cancer Centre Singapore, Singapore. In total 105 FFPE thyroid tissue punches from 63 patients were included in this study. The cohort is composed of five patients with normal thyroid, 28 with multinodular goiter, 10 with follicular thyroid adenoma, five with follicular thyroid carcinoma, and 15 with papillary thyroid carcinoma.

### Pre-treatment and de-crosslinking of FFPE tissue samples

About 1 mg of FFPE tissue was first dewaxed three times by heptane, then rehydrated in a gradient of 100%, 90%, and 75% ethanol (G73537B; Titan, Shanghai, China). The partially rehydrated samples were then transferred into microtubes (MT-96; Pressure Biosciences, Boston, MA) and soaked in 0.1% formic acid (FA) (T-27563; Thermo Fisher Scientific, Waltham, MA) for complete rehydration and acidic hydrolysis for 30 min, under shaking at 600 rpm, 30 °C. The treated FFPE samples were washed using 0.1 M Tris-HCl (pH 10.0) by gentle shaking and spinning down. The supernatant was discarded. 15 µl of 0.1 M Tris-HCl (pH 10.0) was added to cover tissues and the suspension was boiled at 95 °C for 30 min for basic hydrolysis under gentle shaking. Subsequently the sample was fast cooled to 4 °C, topped with 25 µl of lysis buffer containing 6 M urea (U1230; Sigma, St. Louis, MO) and 2 M thiourea (M226; Amresco, India), 0.1 mM NH_4_HCO_3_ (G12990A; Titan) (pH 8.5), and subjected to PCT-assisted tissue lysis and digestion [50].

### PCT-assisted tissue lysis and digestion

After prewashing or de-crosslinking, the FF or FFPE tissues were subjected to PCT-assisted tissue lysis and digestion as described previously [51]. Tissues were lysed in a barocycler NEP2320-45 k (Pressure Biosciences) at the PCT scheme of 30 s high pressure at 45 kpsi plus 10 s ambient pressure, oscillating for 90 cycles at 30 °C. Extracted proteins were reduced and alkylated by incubating with 10 mM Tris (2-carboxyethyl) phosphine (C4706; Sigma, China) and 20 mM iodoacetamide (IAA; I6125; Sigma, China) at 25 °C for 30 min, in darkness, by gently vortexing at 800 rpm in a thermomixer. Afterward, proteins were digested by Lys-C (HLS LYS001C; Hualishi, Beijing, China; enzyme-to-substrate ratio, 1:40) using the PCT scheme of 50 s high pressure at 20 kpsi plus 10 s ambient pressure, oscillating for 45 cycles at 30 °C. This was followed by a tryptic digestion step (HLS TRY001C; Hualishi; enzyme-to-substrate ratio, 1:50) using the PCT scheme of 50 s high pressure at 20 kpsi plus 10 s ambient pressure, oscillating for 90 cycles at 30 °C. Finally, 15 µl of 10% trifluoroacetic acid (TFA) (T/3258/PB05; Thermo Fisher Scientific) was added to each tryptic digest to quench the enzymatic reaction (final concentration of 1% TFA). Peptides were purified by BioPureSPN Midi C18 columns (The Nest Group, Southborough, MA) according to the manufacturer’s protocol. Peptide eluates were then dried under vacuum (CentriVap; LABCONCO, Kansas, MO). Dry peptides were dissolved in 20 µl of water containing 0.1% FA (T-27563; Thermo Fisher Scientific) and 2% acetonitrile (ACN) (34851; Sigma, China) (all MS grade). Peptide concentration was measured using ScanDrop (AnalytikJena, Beijing, China) at A280.

### 1D SDS-PAGE separation at protein level for building DDA library

#### SDS-PAGE separation and peptide preparation in Jimenez lab, the Netherlands

Tissues were lyzed in 1× reducing NuPAGE LDS sample buffer (Invitrogen, Carlsbad, CA), sonicated in a Branson cup-type digital sonifier, centrifuged, and heated for 5 min at 95 °C. Protein lysates were separated on precast 4%–12% gradient gels using the NuPAGE SDS-PAGE system (Invitrogen). Following electrophoresis, gels were fixed in 50% ethanol/3% phosphoric acid solution and stained with Coomassie R-250. Subsequently, gel lanes were cut into 10 bands and each band was cut into ~1 mm^3^ cubes. The gel cubes from each band were transferred into a well of a 96-well filter plate (Eppendorf, Hamburg, Germany) and were washed in 50 mM NH_4_HCO_3_ and 2 × 50 mM NH_4_HCO_3_/50% ACN. Subsequently, gel cubes were reduced for 60 min in 10 mM dithiothreitol (DTT) at 56 °C and alkylated for 45 min in 50 mM IAA (both Sigma, St Louis, MO) in the dark, at 25 °C. After washing with 50 mM NH_4_HCO_3_ and 2 × 50 mM NH_4_HCO_3_/50% ACN, the gel cubes were dried for 10 min in a vacuum centrifuge at 60 °C and subsequently incubated in 50 µl 6.25 ng/µl sequence-grade trypsin (Promega, Madison, WI) in 50 mM NH_4_HCO_3_ at RT overnight. Peptides from each gel band were extracted once using 150 µl 1% FA, and twice using 150 µl 5% FA/50% ACN and were pooled in a 96-deep-well plate and centrifuged to dryness at 60 °C in a vacuum centrifuge and stored at −20 °C. Dried peptide extracts were dissolved in 25 µl loading solvent (0.5% TFA in 4% ACN) containing 2.5 injection equivalent (IE) iRT peptide standard (Biognosys, Schlieren, CH). Finally, 5 µl of peptide extract containing 0.5 IE iRT peptides was injected into the nanoLC system.

#### SDS-PAGE separation and peptide preparation in Guo lab, China

About 200–300 µg of protein was mixed with 3 × SDS sample loading buffer (GenScript Biotech, China) supplemented with 150 mM DTT, and the mixture was boiled at 95 °C for 5 min. Subsequently, 1D gel electrophoresis was performed using 4%–12% gradient SDS-PAGE, after which the gel was removed, washed first with distilled water, and then with the fixing buffer (50% (v/v) ethanol with 5% (v/v) acetic acid in water) at 25 °C for 15 min with gentle agitation to remove excessive SDS. The fixed and washed gel was stained in Coomassie Blue, and then de-stained until the background was clear and protein bands were visible. The gel was rehydrated in distilled water at 25 °C for 10 min with gentle agitation. Subsequently, gel lanes were cut into 10 bands and each band was cut into ~1 mm^3^ cubes, followed by reduction with 10 mM Tris(2-carboxyethyl)phosphine hydrochloride (TCEP) in 25 mM NH_4_HCO_3_ at 25 °C for 1 h, alkylation with 55 mM IAA in 25 mM NH_4_HCO_3_ solution at 25 °C in the dark for 30 min, and sequential digestion with trypsin at a concentration of 12.5 ng/ml at 37 °C overnight (1st digestion for 4 h and 2nd digestion for 12 h). Tryptic-digested peptides were extracted three times using 50% ACN/5% FA and dried under vacuum. Dry peptides were purified by Pierce C18 Spin Tips (Thermo Fisher Scientific).

### Preparation and fractionation of plasma protein samples

Venous blood of each patient was collected in EDTA and anticoagulation proceeded for 9 h. After centrifugation, plasma samples were transferred to a new set of Eppendorf tubes and cold-transported to the proteomics lab at 4 °C within 36 h. Plasma samples were centrifuged again at 300*g* for 5 min at 4 °C to remove cells after arrival at the lab. Supernatant was further centrifuged at 2500*g* for 15 min at 4 °C to remove cell debris and platelets. The final supernatant was stored at −80 °C for further protein digestion.

To remove high abundance plasma proteins, several methods such as SDS-PAGE separation, antibody-depletion of high abundance proteins, and exosome isolation were adopted in this study. For SDS-PAGE fractionation, the entire gel was cut into 12 thin rows, of which four rows with heavily stained protein bands (3 adjacent bands between 45–75 kD, and a band between 25–35 kD) were abandoned for depletion of high abundance proteins. Each of the other remaining 8 rows was subjected to in-gel digestion as described above. In an alternative strategy, the High Select Top 14 Abundant Protein Depletion Resin spin columns (A36370, Thermo Fisher Scientific) was used to deplete high abundance proteins according to the manufacturer’s instructions. After depletion, proteins were further fractionated and digested using 1D SDS-PAGE. To obtain the enriched exosome fraction, an aliquot of 200 µl plasma was collected after centrifuging venous blood for 10 min at 3000*g*, 4 °C. The exosome pellet was further collected after ultracentrifugation at 160,000*g*, 4 °C for 12 h and resuspended in cold PBS for washing. Resuspended exosomes were further centrifuged at 100,000*g*, 4 °C for 70 min. The pellet was redissolved in 150 µl of 2% SDS (S8010; Solarbio, Beijing, China), and was subjected to PCT-assisted sample lysis, undergoing 60 cycles at 20 °C, with 45 kpsi for 50 s and atmosphere pressure for 10 s. After lysis, the exosome protein mixture was precipitated with 80% cold acetone (1000418; Sinopharm Chemical Reagent, China) at −20 °C for 3 h. The suspension was centrifuged at 12,500*g*, 4 °C for 15 min to collect the protein pellet. The protein pellet was redissolved with 200 µl of 1% SDS, followed by 1D SDS-PAGE.

### Strong cation-exchange fractionation at peptide level for building DDA library

For strong cation-exchange (SCX) fractionation, about 1 mg peptides were dissolved in 1 ml of 5 mM KH_2_PO_4_ (G82821D; Titan)/25%ACN (Sigma, 34851, China) (pH3.0), then the peptide solution was loaded onto the well-conditioned SCX SPE cartridge (60108-421; Thermo Fisher Scientific). The cartridge was then rinsed with 5 mM KH_2_PO_4_/25%ACN (pH3.0). Finally, six peptide fractions were collected by eluting the cartridge with 1.5 ml increments of increasing KCl concentration in 5 mM KH_2_PO_4_/25%ACN, *i.e.*, 50 mM, 100 mM, 150 mM, 250 mM, 350 mM, and 500 mM. Each fraction was collected and vacuumed to dryness. Dry peptides and precipitated salts were redissolved in 200 µl of 0.1% TFA (T/3258/PB05; Thermo Fisher Scientific) and subjected to further C18 desalting by BioPureSPN Midi SPE (HEM S18V; Nest Group).

### DDA data acquisition in Jimenez lab

In total 547 DDA raw data files were generated at Jimenez lab. All peptides were prepared via 1D SDS-PAGE. Peptides were separated by an Ultimate 3000 nanoLC-MS/MS system (Dionex LC-Packings, Amsterdam, The Netherlands) equipped with a 40 cm × 75 μm inner diameter (ID) fused silica column custom packed with 1.9 μm 120 Å ReproSil Pur C18 aqua (Dr Maisch GMBH, Ammerbuch-Entringen, Germany). After injection, peptides were trapped at 10 μl/min on a 10 mm × 100 μm ID trap column packed with 5 μm 120 Å ReproSil Pur C18 aqua in 0.1% FA. Peptides were separated at 300 nl/min along a 90-min 10%–40% linear LC gradient (buffer A: 0.1% FA; buffer B: 80% ACN, 0.1% FA) (130 min inject-to-inject in total). Eluting peptides were ionized at a potential of +2 kV into a Q Exactive mass spectrometer (Thermo Fisher Scientific, Bremen, Germany). Intact masses were measured at resolution 70,000 (at m/z of 200) in the Orbitrap using an automatic gain control (AGC) target value of 3E6 charges and an S-lens setting of 60. The top 10 peptide signals (charge state 2+ and higher) were submitted to MS/MS in the higher-energy collision (HCD) cell (1.6 amu isolation width, 25% normalized collision energy). MS/MS spectra were acquired at resolution 17,500 (at m/z of 200) in the Orbitrap using an AGC target value of 1E6 charges, a max injection time (IT) of 80 ms, and an underfill ratio of 0.1%. Dynamic exclusion was applied with a repeat count of 1 and an exclusion time of 30 s.

### DDA data acquisition in Guo lab

In total 549 DDA raw data files were generated in Guo lab. Biognosys-11 iRT peptides (Biognosys) were spiked into peptide samples at the final concentration of 10% prior to MS injection for RT calibration. Peptides were separated by Ultimate 3000 nanoLC-MS/MS system (Dionex LC-Packings) equipped with a 15 cm × 75 μm ID fused silica column (National Institute of Biological Sciences, Beijing, China) packed with 1.9 μm 100 Å C18. After injection, peptides were trapped at 6 μl/min on a 20 mm × 75 μm ID trap column (Thermo Fisher Scientific, Waltham, MA) packed with 3 μm 100 Å C18 aqua in 0.1% FA. Peptides were separated along a 120-min 3%–25% linear LC gradient (buffer A: 2% ACN, 0.1% FA; buffer B: 98% ACN, 0.1% FA) at the flowrate of 300 nl/min (148 min inject-to-inject in total). Eluted peptides were ionized at a potential of +1.8 kV into a Q-Exactive HF MS (Thermo Fisher Scientific, Waltham, MA). Intact masses were measured at resolution 60,000 (at m/z of 200) in the Orbitrap using an AGC target value of 3E6 charges and an S-lens setting of 50. The top 20 peptide signals (charge-states 2+ and higher) were submitted to MS/MS in the HCD cell (1.6 amu isolation width, 27% normalized collision energy). MS/MS spectra were acquired at resolution 30,000 (at m/z of 200) in the Orbitrap using an AGC target value of 1E5 charges, a max IT of 80 ms, and an underfill ratio of 0.1%. Dynamic exclusion was applied with a repeat count of 1 and an exclusion time of 30 s.

### DIA data acquisition in Guo lab

The LC configuration for DIA data acquisition is as the same as for DDA data acquisition with slight modifications. Biognosys-11 iRT peptides (Biognosys) were spiked into peptide samples at the final concentration of 10% prior to MS injection for RT calibration. Peptides were separated at 300 nl/min in a 3%–25% linear gradient (buffer A: 2% ACN, 0.1% FA; buffer B: 98% ACN, 0.1% FA) in 60 min (75 min inject-to-inject in total) for prostate samples and 20 min (35 min inject-to-inject in total) for plasma samples. Eluted peptides were ionized at a potential of +1.8 kV into a Q-Exactive HF mass spectrometer (Thermo Fisher Scientific, Waltham, MA). A full MS scan was acquired by analyzing 390–1010 m/z at resolution 60,000 (at m/z of 200) in the Orbitrap using an AGC target value of 3E6 charges and maximum IT of 80 ms. After the MS scan, 24 MS/MS scans were acquired, each with a 30,000 resolution at a m/z of 200, AGC target 1E6 charges, and normalized collision energy as 27%, with the default charge state set to 2 and maximum IT set to auto. The cycle of 24 MS/MS scans (center of isolation window) with three kinds of wide isolation window are as follows (m/z): 410, 430, 450, 470, 490, 510, 530, 550, 570, 590, 610, 630, 650, 670, 690, 710, 730, 750, 770, 790, 820, 860, 910, and 970. The entire cycle of MS and MS/MS scans acquisition took roughly 3 s and was repeated throughout the LC/MS/MS analysis.

### DIA data analysis using OpenSWATH and TRIC

Briefly, DIA raw data files were converted in profile mode to mzXML using msconvert and analyzed using OpenSWATH (2.0.0) [14] as described previously [13]. Retention time extraction window was set as 600 s (for 60 min LC) or 350 s (for 20 min LC), and m/z extraction was performed with 0.03 Da tolerance. Retention time was then calibrated using both SiRT and CiRT peptides. Peptide precursors that were identified by OpenSWATH and pyprophet with d_score > 0.01 were used as inputs for TRIC [52]. For each protein, the median MS2 intensity value of peptide precursor fragments that were detected to belong to the protein was used to represent the protein abundance.

### Terms for protein identification

In this paper, the term “protein group” indicates (i) one protein that is identified by a set of peptides that are not included in any other protein group, and (ii) a group of proteins sharing the same set or subset of identified peptides. Proteins identified from Swiss-Prot protein sequence database (*i.e.*, one manually inspected protein sequence per gene symbol, excluding isoforms, splicing variants, and theoretical protein sequences) are called “Swiss-Prot proteins”.

### Validation of representative proteins using PRM

PRM quantification strategy was used to further validate proteins that were measured by DIA quantification above. Biognosys-11 iRT peptides (Biognosys) were spiked into peptide samples at the final concentration of 10% prior to MS injection for RT calibration. Peptides were separated at 300 nl/min along a 60 min 7%–35% linear LC gradient (buffer A: 100% water, 0.1% FA; buffer B: 80% ACN, 0.1% FA). The Orbitrap Fusion Lumos Tribrid mass spectrometer (Thermo Fisher Scientific, Waltham, MA) was operated in the MS/MS mode with time-scheduled acquisition for 100 peptides in a +/− 5 min retention time window. The individual isolation window was set at 1.2 Th. The full MS mode was measured at resolution 60,000 at the m/z of 200 in the Orbitrap, with AGC target value of 4E5 and maximum IT of 50 ms. Target ions were submitted to MS/MS in the HCD cell (1.2 amu isolation width, 30% normalized collision energy). MS/MS spectra were acquired at resolution 30,000 (at m/z of 200) in the Orbitrap using AGC target value of 1E5 and maximum IT of 100 ms.

## Ethical statement

Ethics approvals for this study were obtained from the Ethics Committee or Institutional Review Board of each participating institution.

## Data availability

Computational pipeline as a Docker container (user could get images with the command ‘docker pull guomics/dphl:v02’) and DPHL (tsv flat file, splib, pepidx, and spidx library files in DLBCL sub project), all the DDA files, DIA-MS Data files, original peptides, and protein results are deposited in iProX (iProX: IPX0001400000) and can be accessed at https://www.iprox.org/page/project.html?id=IPX0001400000.

## CRediT author statement

**Tiansheng Zhu:** Conceptualization, Methodology, Project administration, Supervision, Writing - original draft, Writing - review & editing, Data curation, Software, Formal analysis. **Yi Zhu:** Project administration, Supervision, Writing - original draft, Writing - review & editing, Visualization, Resources, Funding acquisition. **Yue Xuan:** Resources. **Huanhuan Gao:** Resources. **Xue Cai:** Resources. **Sander R. Piersma:** Resources. **Thang V. Pham:** Visualization. **Tim Schelfhorst:** Resources. **Richard R.G.D. Haas:** . **Irene V. Bijnsdorp:** Visualization, Resources. **Rui Sun:** Resources. **Liang Yue:** Resources. **Guan Ruan:** Visualization. **Qiushi Zhang:** Visualization. **Mo Hu:** Resources. **Yue Zhou:** Resources. **Winan J. Van Houdt:** Resources. **Tessa Y.S. Lelarge:** Resources. **Jacqueline Cloos:** Resources. **Anna Wojtuszkiewicz:** Resources. **Danijela Koppers-Lalic:** Resources. **Franziska Böttger:** Resources. **Chantal Scheepbouwer:** Resources. **Ruud H. Brakenhoff:** Resources. **Geert J.L.H. van Leenders:** Resources. **Jan N.M. Ijzermans:** Resources. **John W.M. Martens:** Resources. **Renske D.M. Steenbergen:** Resources. **Nicole C. Grieken:** Resources. **Sathiyamoorthy Selvarajan:** Resources. **Sangeeta Mantoo:** Resources. **Sze S. Lee:** Resources. **Serene J.Y. Yeow:** Resources. **Syed M.F. Alkaff:** Resources. **Nan Xiang:** Resources. **Yaoting Sun:** Resources. **Xiao Yi:** Data curation, Resources. **Shaozheng Dai:** Visualization. **Wei Liu:** Resources. **Tian Lu:** Resources. **Zhicheng Wu:** Visualization. **Xiao Liang:** Resources. **Man Wang:** Resources. **Yingkuan Shao:** Resources. **Xi Zheng:** Resources. **Kailun Xu:** Resources. **Qin Yang:** Resources. **Yifan Meng:** Resources. **Cong Lu:** Resources. **Jiang Zhu:** Resources. **Jin’e Zheng:** Resources. **Bo Wang:** Resources. **Sai Lou:** Resources. **Yibei Dai:** Resources. **Chao Xu:** Software. **Chenhuan Yu:** Resources. **Huazhong Ying:** Resources. **Tony K. Lim:** Resources. **Jianmin Wu:** Resources. **Xiaofei Gao:** Resources. **Zhongzhi Luan:** Software. **Xiaodong Teng:** Resources. **Peng Wu:** Resources. **Shi’ang Huang:** Resources. **Zhihua Tao:** Resources. **Narayanan G. Iyer:** Resources. **Shuigeng Zhou:** Project administration, Supervision. **Wenguang Shao:** Software. **Henry Lam:** Software, Resources. **Ding Ma:** Resources. **Jiafu Ji:** Resources. **Oi L. Kon:** Resources. **Shu Zheng:** Resources. **Ruedi Aebersold:** . **Connie R. Jimenez:** Project administration, Supervision, Resources. **Tiannan Guo:** Conceptualization, Methodology, Project administration, Supervision, Writing - original draft, Writing - review & editing, Funding acquisition. All authors read and approved the final manuscript.

## Competing interests

The research group of Tiannan Guo is partly supported by Thermo Fisher Scientific and Pressure Biosciences, which provided access to advanced sample preparation instrumentation. Yue Xuan, Mo Hu, and Yue Zhou were employees of Thermo Fisher Scientific during this project. The remaining authors declare no competing interests.

## Declaration of Competing Interest

The research group of Tiannan Guo is partly supported by Thermo Fisher Scientific and Pressure Biosciences, which provided access to advanced sample preparation instrumentation. Yue Xuan, Mo Hu, and Yue Zhou were employees of Thermo Fisher Scientific during this project. The remaining authors declare no competing interests.

## Supplementary data

Supplementary Figure S1 Comparison of protein sequence coverage between DPHL and PHL. Violin plot (A) and density plot (B) show sequence coverage of the proteins identified by DPHL and PHL.

Supplementary Figure S2 Accumulated datasets in DPHL. A. Number of transition groups (peptide precursors) in accumulated datasets in DPHL. The number of peptide precursors in DPHL increases cumulatively when adding datasets from various tissue types. B. Cumulative protein counts in DPHL. The number of proteins gets saturated as the number of raw data files increases. C. Cumulative peptide precursor and protein counts from pancreatic tissue samples based on 100 DDA files. Upper panel: number of peptide precursors; lower panel: number of proteins identified. D. Cumulative peptide precursor and protein counts from 179 plasma samples. Upper panel: number of peptide precursors; lower panel: number of proteins identified.

Supplementary Figure S3 GO enrichment for all proteins identified in DPHL. In total 18 GO groups were enriched. A. Dot plot of GO enrichment. Gene ratio is calculated as the number of genes in DPHL against the number of all genes with the same GO term. B. Bar plot of GO enrichment. Length of bar indicates number of proteins is indicated by circle size or bar length; adjusted P values are color coded.

Supplementary Figure S4 GO enrichment for all proteins identified in DPHL. In total 18 GO groups were enriched. A. Dot plot of GO enrichment. Gene ratio is calculated as the number of genes in DPHL against the number of all genes with the same GO term. B. Bar plot of GO enrichment. Length of bar indicates number of proteins is indicated by circle size or bar length; adjusted *P* values are color coded.

Supplementary Figure S5 Protein kinases identified in DPHL. A. Bar charts showing the number of proteins (left, blue) and the kinases (right, red) identified in the DPHL library. B. Overlap of kinases for different tissue types. C. Venn diagrams showing the comparison of DPHL with Swiss-Prot and the kinome. DPHL covers about 70% kinases in the Swiss-Prot database.

Supplementary Figure S6 542 TF proteins identified in DPHL using the Human Transcription Factors database (http://humantfs.ccbr.utoronto.ca/) as a reference.

Supplementary Figure S7 Identified contents in the DLBCL cohort. Peptide precursors (mean: 4313, min: 3855; max: 4728; A), protein groups (mean: 332, min: 309; max: 363; B), and proteins (mean: 195, min: 184; max: 213; C) were identified in 37 plasma samples from the DLBCL cohort, including 19 DLBCL patients and 18 HC subjects.

Supplementary Figure S8 Peak area for peptide LFGGNFAHQASVAR of TPP1 across 73 PRM runs in the PCa cohort. Relative abundance of TPP1 across 73 PRM runs in the PCa cohort was quantified using Skyline, with one representative example shown for LFGGNFAHQASVAR (m/z 737.88, 2+). A. XIC peak areas of the precursor ions obtained at MS1 level. B. XIC peak areas of fragment ions obtained at MS2 level.

Supplementary Figure S9 PRM validation of CRP and SAA1 in the DLBCL cohort. Two best flying peptides were selected to quantify CRP (upper panels); one best flying peptide was selected for SAA1 (lower panels). Each box represents the information for one peptide. Box plots show the log2 MS2 intensity of the respective peptides across 37 plasma samples from the DLBCL cohort, including 19 DLBCL patients and 18 HC subjects (on the left), while XICs depict a representative peak group for each peptide from sample s8 (on the right). P values are computed using Student’s *t* test.

Supplementary Figure S10 Peak area for peptide ESDTSYVSLK of CRP across 37 plasma samples in the DLBCL cohort. The XIC peaks of the precursor ions at MS1 level (A) and fragment ions at MS2 level (B) for peptide ESDTSYVSLK (m/z 564.77, 2+) of CRP (P02741) were obtained from the DLBCL cohort, including 19 DLBCL patients and 18 HC subjects.

Supplementary Table S1 Tissue and peptide samples used for the DPHL project. A. Summary information of the tissue samples used for the DPHL project. B. Detailed information of the peptide samples used for the DPHL project.

Supplementary Table S2 CiRT data in detail. A. Detailed information of CiRT peptides and their transitional groups for 16 tissue types. B. The list of CiRT peptides for each tissue type. Tissue types including bladder, cervix, colorectal (colon & rectum), DLBCL (Diffuse Large B Cell Lymphoma), stomach (gastric), brain (glioblastoma multiforme), liver, k562, leukemia, lung, PDAC (pancreas, Pancreatic Ductal Adenocarcinoma), plasma, prostate, sarcoma, thyroid, breast (tnbc, triple negative breast cancer).

Supplementary Table S3 DIA data for PCa cohort. A. Clinical information for PCa patients analyzed in the DIA experiments. B. Sample information for the 24 samples. C. Protein matrix generated using CiRT strategy. D. Protein matrix generated using SiRT strategy. E. Differentially expressed proteins using CiRT. F. Differentially expressed proteins using SiRT. Note: Patient A1/P7, A2/P49, A3/P51, PN-4: each had one biological replicate.

Supplementary Table S4 PCa biomarker discovery. A. 400 proteins selected by random forest. B. DAVID enrichment analysis. C. 86 shortlisted proteins.

Supplementary Table S5 DIA data for DLBCL cohort . A. Clinical information for DLBCL patients (19 DLBCL patients and 18 healthy controls.). B. Sample information. C. Protein matrix. D. Differentially expressed proteins. E. Peptide Matrix for reproducibility analysis.

Supplementary Table S6 PRM data for PCa cohort. A. Clinical information for PCa patients recruited in the PRM study. B. Peptide sample information for PRM MS/MS analysis. C. Peptide intensity calculated for each protein.

Supplementary Table S7 PRM data for DLBCL cohort. A. Clinical information for the 37 patients (19 DLBCL and 18 healthy controls). B. Peptide sample information for PRM MS/MS analysis. C. Peptide intensity calculated for each protein.

Supplementary File S1 Comparison of DDA files acquired from the Guo lab and the Jimenez lab.

Supplementary File S2 TraML format of bladder CiRT.

Supplementary File S3 TraML format of cervix CiRT.

Supplementary File S4 TraML format of colon & rectum CiRT.

Supplementary File S5 TraML format of DLBCL CiRT.

Supplementary File S6 TraML format of stomach CiRT.

Supplementary File S7 TraML format of brain CiRT.

Supplementary File S8 TraML format of liver CiRT.

Supplementary File S9 TraML format of K562 CiRT.

Supplementary File S10 TraML format of leukemia CiRT.

Supplementary File S11 TraML format of lung CiRT.

Supplementary File S12 TraML format of pancreas CiRT.

Supplementary File S13 TraML format of plasma CiRT.

Supplementary File S14 TraML format of prostate CiRT.

Supplementary File S15 TraML format of sarcoma CiRT.

Supplementary File S16 TraML format of thyroid CiRT.

Supplementary File S17 TraML format of breast CiRT.

Supplementary File S18 Computational workflow for building DPHL spectral library for DIA MS/MS.

Supplementary File S19 Practical guide using test data as an example.

## References

[1] Schubert O.T., Gillet L.C., Collins B.C., Navarro P., Rosenberger G., Wolski W.E. (2015). Building high-quality assay libraries for targeted analysis of SWATH MS data. Nat Protoc.

[2] Sandhu C., Qureshi A., Emili A. (2018). Panomics for precision medicine. Trends Mol Med.

[3] Aronson S.J., Rehm H.L. (2015). Building the foundation for genomics in precision medicine. Nature.

[4] Yang J.Y.C., Sarwal M.M. (2017). Transplant genetics and genomics. Nat Rev Genet.

[5] Zhang B., Wang J., Wang X., Zhu J., Liu Q., Shi Z. (2014). Proteogenomic characterization of human colon and rectal cancer. Nature.

[6] Bosch L.J.W., de Wit M., Pham T.V., Coupe V.M.H., Hiemstra A.C., Piersma S.R. (2017). Novel stool-based protein biomarkers for improved colorectal cancer screening: a case-control study. Ann Intern Med.

[7] Mertins P., Mani D.R., Ruggles K.V., Gillette M.A., Clauser K.R., Wang P. (2016). Proteogenomics connects somatic mutations to signalling in breast cancer. Nature.

[8] Zhang H., Liu T., Zhang Z., Payne S.H., Zhang B., McDermott J.E. (2016). Integrated proteogenomic characterization of human high-grade serous ovarian cancer. Cell.

[9] Ge S., Xia X., Ding C., Zhen B., Zhou Q., Feng J. (2018). A proteomic landscape of diffuse-type gastric cancer. Nat Commun.

[10] Zhu Y.i., Guo T. (2018). Towards a one-stop solution for large-scale proteomics data analysis. Sci China Life Sci.

[11] Cominetti O., Nunez Galindo A., Corthesy J., Valsesia A., Irincheeva I., Kussmann M. (2018). Obesity shows preserved plasma proteome in large independent clinical cohorts. Sci Rep.

[12] Gillet L.C., Navarro P., Tate S., Rost H., Selevsek N., Reiter L. (2012). Targeted data extraction of the MS/MS spectra generated by data-independent acquisition: a new concept for consistent and accurate proteome analysis. Mol Cell Proteomics.

[13] Guo T., Kouvonen P., Koh C.C., Gillet L.C., Wolski W.E., Rost H.L. (2015). Rapid mass spectrometric conversion of tissue biopsy samples into permanent quantitative digital proteome maps. Nat Med.

[14] Rost H.L., Rosenberger G., Navarro P., Gillet L., Miladinovic S.M., Schubert O.T. (2014). OpenSWATH enables automated, targeted analysis of data-independent acquisition MS data. Nat Biotechnol.

[15] Navarro P., Kuharev J., Gillet L.C., Bernhardt O.M., MacLean B., Rost H.L. (2016). A multicenter study benchmarks software tools for label-free proteome quantification. Nat Biotechnol.

[16] Tsou C.C., Avtonomov D., Larsen B., Tucholska M., Choi H., Gingras A.C. (2015). DIA-Umpire: comprehensive computational framework for data-independent acquisition proteomics. Nat Methods.

[17] Li Y., Zhong C.Q., Xu X., Cai S., Wu X., Zhang Y. (2015). Group-DIA: analyzing multiple data-independent acquisition mass spectrometry data files. Nat Methods.

[18] MacLean B., Tomazela D.M., Shulman N., Chambers M., Finney G.L., Frewen B. (2010). Skyline: an open source document editor for creating and analyzing targeted proteomics experiments. Bioinformatics.

[19] Bruderer R., Bernhardt O.M., Gandhi T., Miladinovic S.M., Cheng L.Y., Messner S. (2015). Extending the limits of quantitative proteome profiling with data-independent acquisition and application to acetaminophen-treated three-dimensional liver microtissues. Mol Cell Proteomics.

[20] Rosenberger G., Koh C.C., Guo T., Rost H.L., Kouvonen P., Collins B.C. (2014). A repository of assays to quantify 10,000 human proteins by SWATH-MS. Sci Data.

[21] Rosenberger G., Bludau I., Schmitt U., Heusel M., Hunter C.L., Liu Y. (2017). Statistical control of peptide and protein error rates in large-scale targeted data-independent acquisition analyses. Nat Methods.

[22] Liu Y., Buil A., Collins B.C., Gillet L.C., Blum L.C., Cheng L.Y. (2015). Quantitative variability of 342 plasma proteins in a human twin population. Mol Syst Biol.

[23] Guo T., Li L., Zhong Q., Rupp N.J., Charmpi K., Wong C.E. (2018). Multi-region proteome analysis quantifies spatial heterogeneity of prostate tissue biomarkers. Life Sci Alliance.

[24] Zhu Y., Zhu J., Lu C., Zhang Q., Xie W., Sun P. (2018). Identification of protein abundance changes in hepatocellular carcinoma tissues using PCT-SWATH. Proteomics Clin Appl.

[25] Muntel J., Xuan Y., Berger S.T., Reiter L., Bachur R., Kentsis A. (2015). Advancing urinary protein biomarker discovery by data-independent acquisition on a quadrupole-orbitrap mass spectrometer. J Proteome Res.

[26] Meyer J.G., Schilling B. (2017). Clinical applications of quantitative proteomics using targeted and untargeted data-independent acquisition techniques. Expert Rev Proteomics.

[27] Chambers M.C., Maclean B., Burke R., Amodei D., Ruderman D.L., Neumann S. (2012). A cross-platform toolkit for mass spectrometry and proteomics. Nat Biotechnol.

[28] Li D., Fu Y., Sun R., Ling C.X., Wei Y., Zhou H. (2005). pFind: a novel database-searching software system for automated peptide and protein identification via tandem mass spectrometry. Bioinformatics.

[29] Lam H., Deutsch E.W., Eddes J.S., Eng J.K., King N., Stein S.E. (2007). Development and validation of a spectral library searching method for peptide identification from MS/MS. Proteomics.

[30] Parker S.J., Rost H., Rosenberger G., Collins B.C., Malmstrom L., Amodei D. (2015). Identification of a set of conserved eukaryotic internal retention time standards for data-independent acquisition mass spectrometry. Mol Cell Proteomics.

[31] Escher C., Reiter L., MacLean B., Ossola R., Herzog F., Chilton J. (2012). Using iRT, a normalized retention time for more targeted measurement of peptides. Proteomics.

[32] Cox J., Mann M. (2008). MaxQuant enables high peptide identification rates, individualized p.p.b.-range mass accuracies and proteome-wide protein quantification. Nat Biotechnol.

[33] Eid S., Turk S., Volkamer A., Rippmann F., Fulle S. (2017). KinMap: a web-based tool for interactive navigation through human kinome data. BMC Bioinformatics.

[34] Lambert S.A., Jolma A., Campitelli L.F., Das P.K., Yin Y., Albu M. (2018). The human transcription factors. Cell.

[35] van der Maaten L. (2014). Accelerating t-SNE using tree-based algorithms. J Mach Learn Res.

[36] Tripathi S., Pohl M.O., Zhou Y., Rodriguez-Frandsen A., Wang G., Stein D.A. (2015). Meta- and orthogonal integration of influenza “OMICs” data defines a role for UBR4 in virus budding. Cell Host Microbe.

[37] Shurbaji M.S., Kalbfleisch J.H., Thurmond T.S. (1996). Immunohistochemical detection of a fatty acid synthase (OA-519) as a predictor of progression of prostate cancer. Hum Pathol.

[38] Xin H., Liu D., Wan M., Safari A., Kim H., Sun W. (2007). *TPP1* is a homologue of ciliate TEBP-beta and interacts with POT1 to recruit telomerase. Nature.

[39] Nandakumar J., Bell C.F., Weidenfeld I., Zaug A.J., Leinwand L.A., Cech T.R. (2012). The TEL patch of telomere protein *TPP1* mediates telomerase recruitment and processivity. Nature.

[40] Sexton A.N., Regalado S.G., Lai C.S., Cost G.J., O'Neil C.M., Urnov F.D. (2014). Genetic and molecular identification of three human *TPP1* functions in telomerase action: recruitment, activation, and homeostasis set point regulation. Genes Dev.

[41] Mocellin S., Pooley K.A., Nitti D. (2013). Telomerase and the search for the end of cancer. Trends Mol Med.

[42] Heaphy C.M., Meeker A.K. (2011). The potential utility of telomere-related markers for cancer diagnosis. J Cell Mol Med.

[43] Qian X., Li C., Pang B., Xue M., Wang J., Zhou J. (2012). Spondin-2 (*SPON2*), a more prostate-cancer-specific diagnostic biomarker. PLoS One.

[44] Lucarelli G., Rutigliano M., Bettocchi C., Palazzo S., Vavallo A., Galleggiante V. (2013). Spondin-2, a secreted extracellular matrix protein, is a novel diagnostic biomarker for prostate cancer. J Urol.

[45] Steuber T., O'Brien M.F., Lilja H. (2008). Serum markers for prostate cancer: a rational approach to the literature. Eur Urol.

[46] Cao Y., Shi Y.X., Chen J.O., Tan Y.T., Cai Y.C., Luo H.Y. (2012). Serum C-reactive protein as an important prognostic variable in patients with diffuse large B cell lymphoma. Tumour Biol.

[47] Tzankov A., Pehrs A.C., Zimpfer A., Ascani S., Lugli A., Pileri S. (2003). Prognostic significance of CD44 expression in diffuse large B cell lymphoma of activated and germinal centre B cell-like types: a tissue microarray analysis of 90 cases. J Clin Pathol.

[48] Ling J.Y., Sun X.F., Zhang X., Zhen Z.J., Xia Y., Luo W.B. (2008). Dynamic changes of serum proteomic spectra in patients with non-Hodgkin's lymphoma (NHL) before and after chemotherapy and screening of candidate biomarkers for NHL. Chin J Cancer.

[49] Jimenez C.R., Zhang H., Kinsinger C.R., Nice E.C. (2018). The cancer proteomic landscape and the HUPO Cancer Proteome Project. Clin Proteom.

[50] Zhu Y., Weiss T., Zhang Q., Sun R., Wang B., Yi X. (2019). High-throughput proteomic analysis of FFPE tissue samples facilitates tumor stratification. Mol Oncol.

[51] Zhu Y., Guo T. (2018). High-throughput proteomic analysis of fresh-frozen biopsy tissue samples using pressure cycling technology coupled with SWATH mass spectrometry. Methods Mol Biol.

[52] Rost H.L., Liu Y., D'Agostino G., Zanella M., Navarro P., Rosenberger G. (2016). TRIC: an automated alignment strategy for reproducible protein quantification in targeted proteomics. Nat Methods.

