# Supplementary figures and images for: DPHL: A DIA Pan-human Protein Mass Spectrometry Library for Robust Biomarker Discovery
Source: Genomics Proteomics Bioinformatics. 2020 Aug 12;18(2):104–19. doi: 10.1016/j.gpb.2019.11.008 (PMC7646093; doi:10.1016/j.gpb.2019.11.008)

A

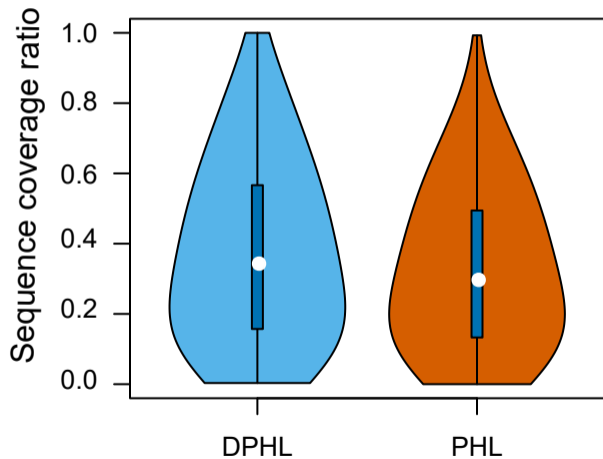

B

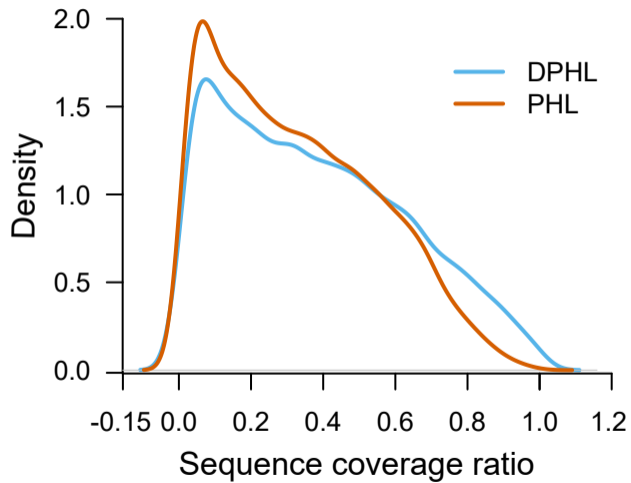

Supplement: Supplementary Figure S1 — Comparison of protein sequence coverage between DPHL and PHL. Violin plot (A) and density plot (B) show sequence coverage of the proteins identified by DPHL and PHL. [file mmc1.pdf]

A Dotplot of GO enrichment

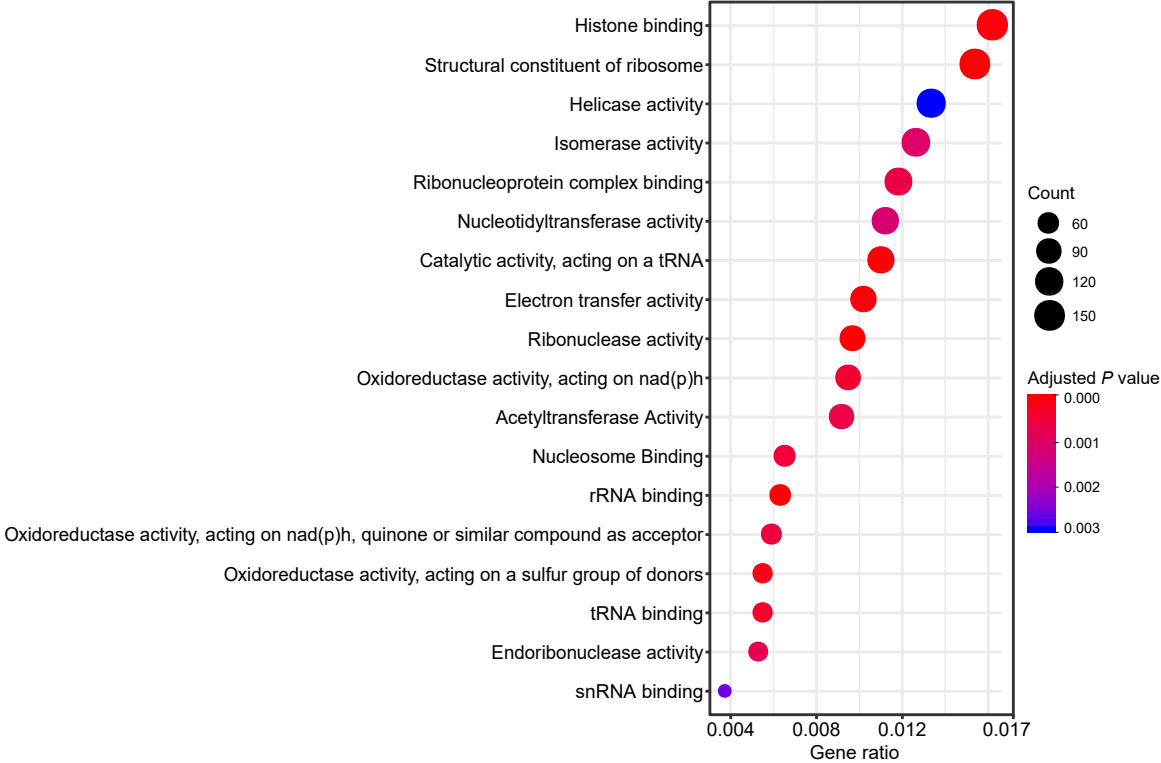

B Barplot of GO enrichment

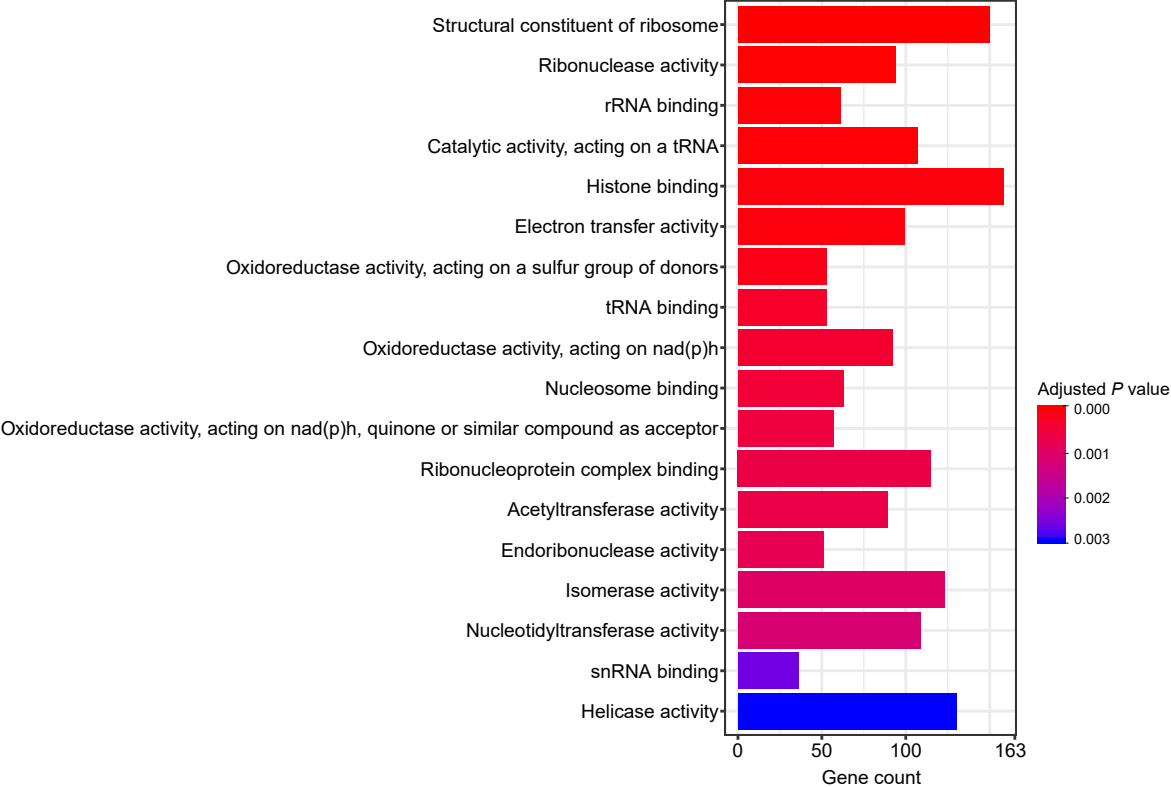

Supplement: Supplementary Figure S3 — GO enrichment for all proteins identified in DPHL. In total 18 GO groups were enriched. A. Dot plot of GO enrichment. Gene ratio is calculated as the number of genes in DPHL against the number of all genes with the same GO term. B. Bar plot of GO enrichment. Length of bar indicates number of proteins is indicated by circle size or bar length; adjusted P values are color coded. [file mmc3.pdf]

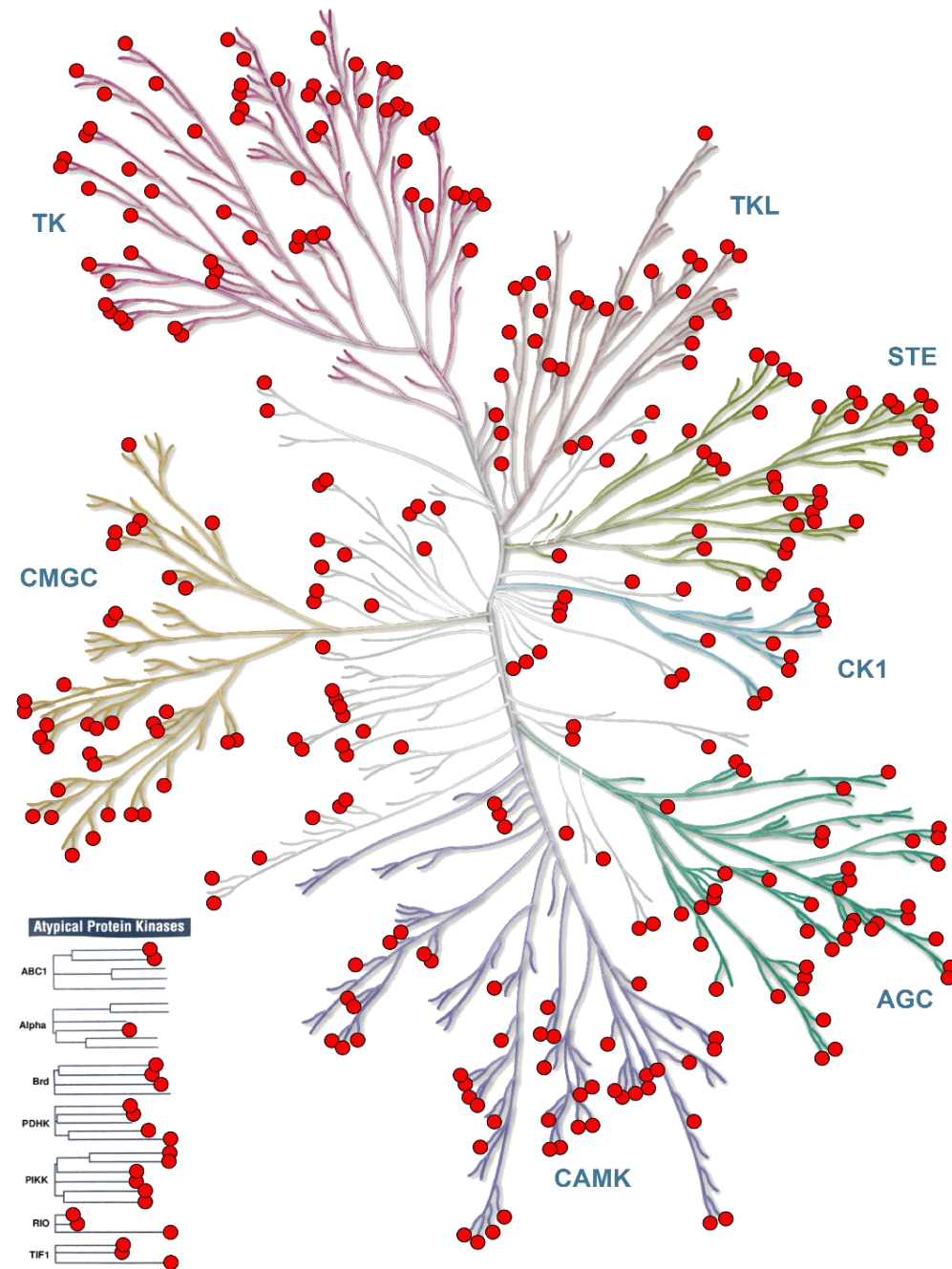

Supplement: Supplementary Figure S4 — GO enrichment for all proteins identified in DPHL. In total 18 GO groups were enriched. A. Dot plot of GO enrichment. Gene ratio is calculated as the number of genes in DPHL against the number of all genes with the same GO term. B. Bar plot of GO enrichment. Length of bar indicates number of proteins is indicated by circle size or bar length; adjusted P values are color coded. [file mmc4.pdf]

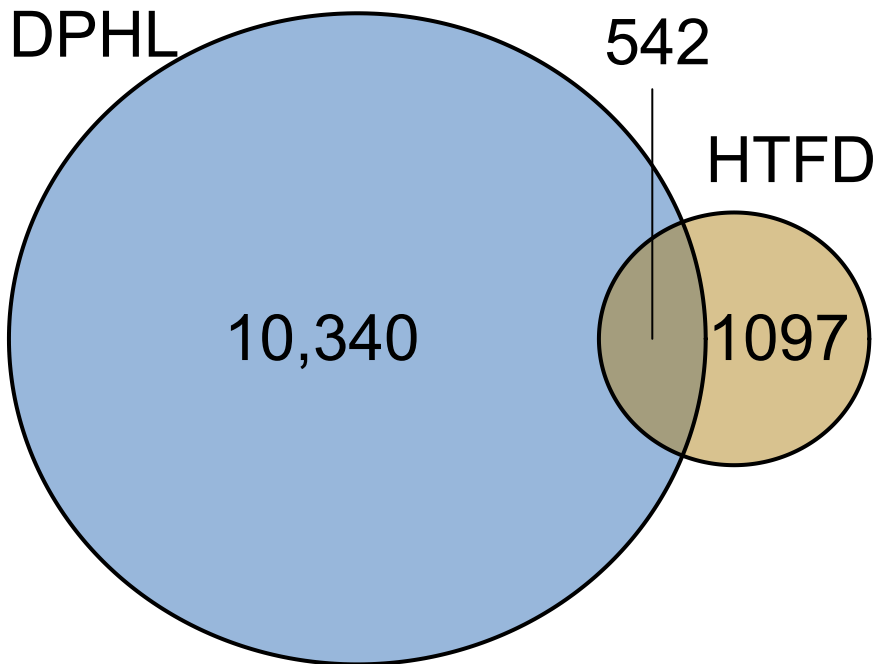

Supplement: Supplementary Figure S6 — 542 TF proteins identified in DPHL using the Human Transcription Factors database (http://humantfs.ccbr.utoronto.ca/) as a reference. [file mmc6.pdf]

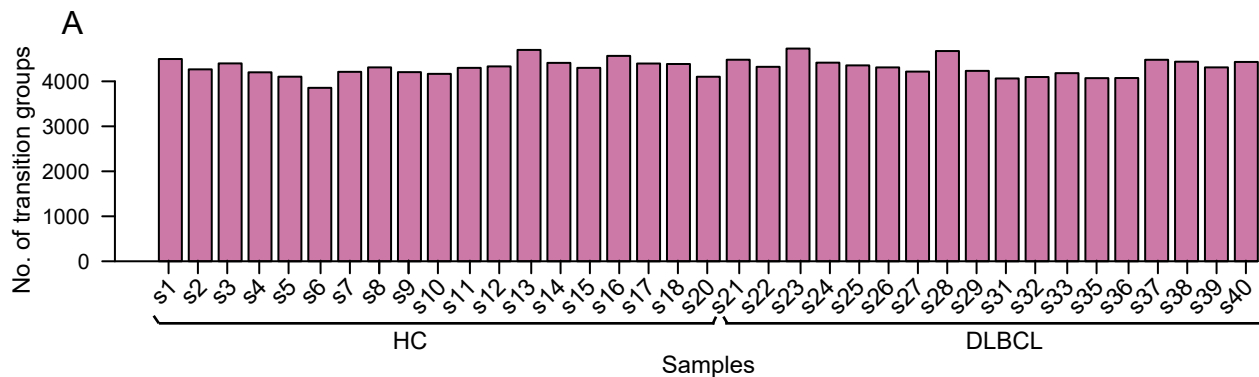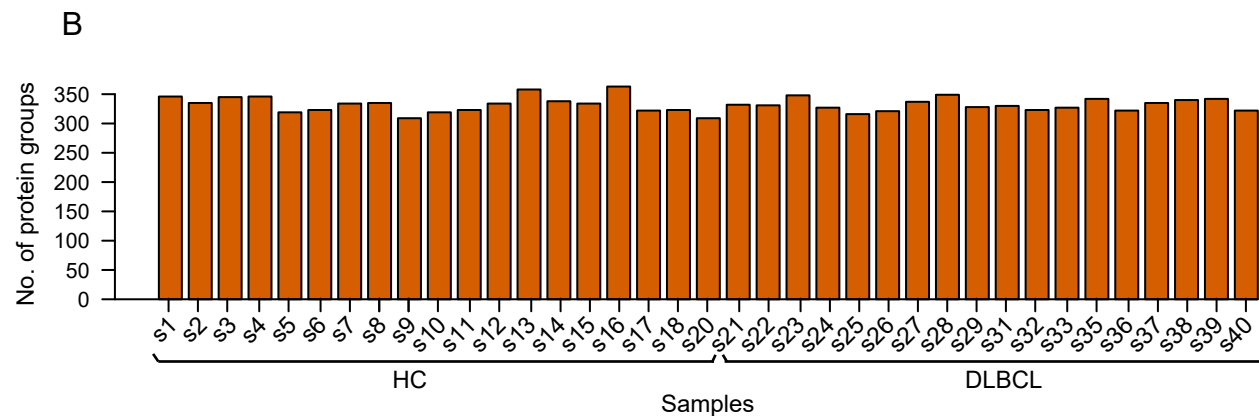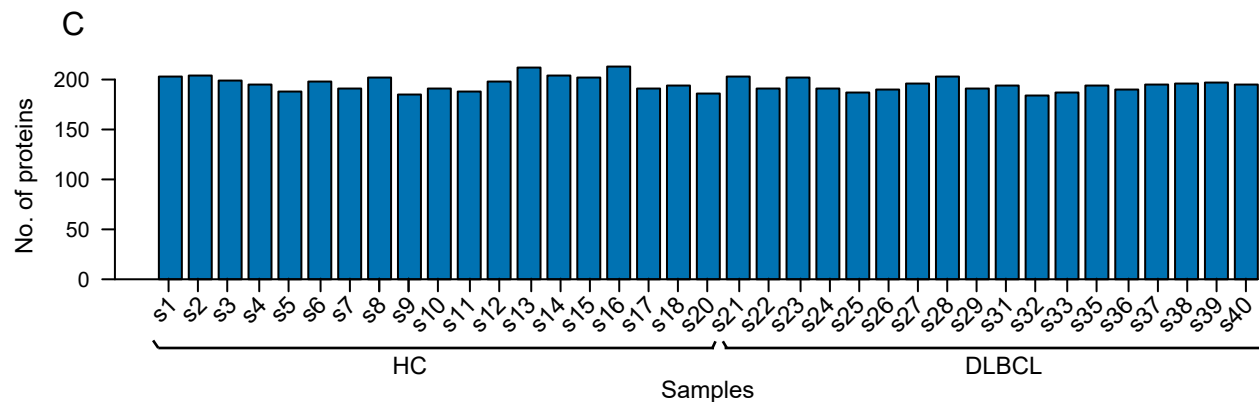

Supplement: Supplementary Figure S7 — Identified contents in the DLBCL cohort. Peptide precursors (mean: 4313, min: 3855; max: 4728; A), protein groups (mean: 332, min: 309; max: 363; B), and proteins (mean: 195, min: 184; max: 213; C) were identified in 37 plasma samples from the DLBCL cohort, including 19 DLBCL patients and 18 HC subjects. [file mmc7.pdf]

## A XIC peak areas of precursor ions at MS1 level

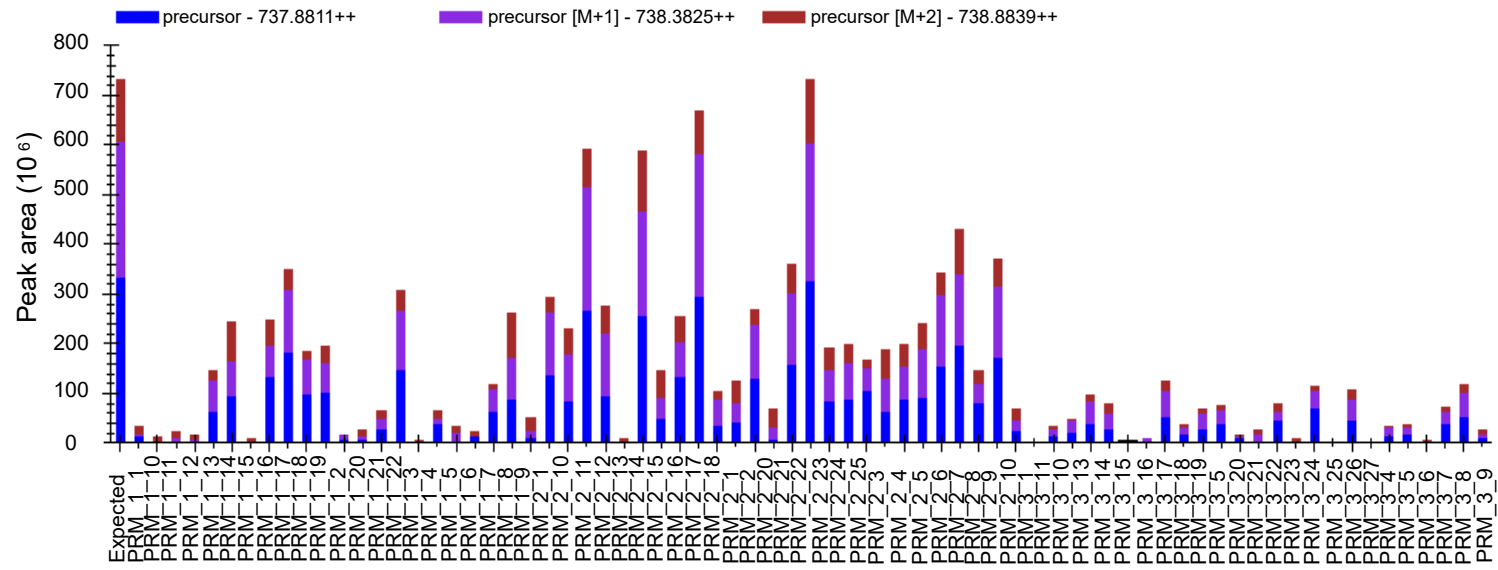

## B XIC peak areas of fragment ions at MS2 level

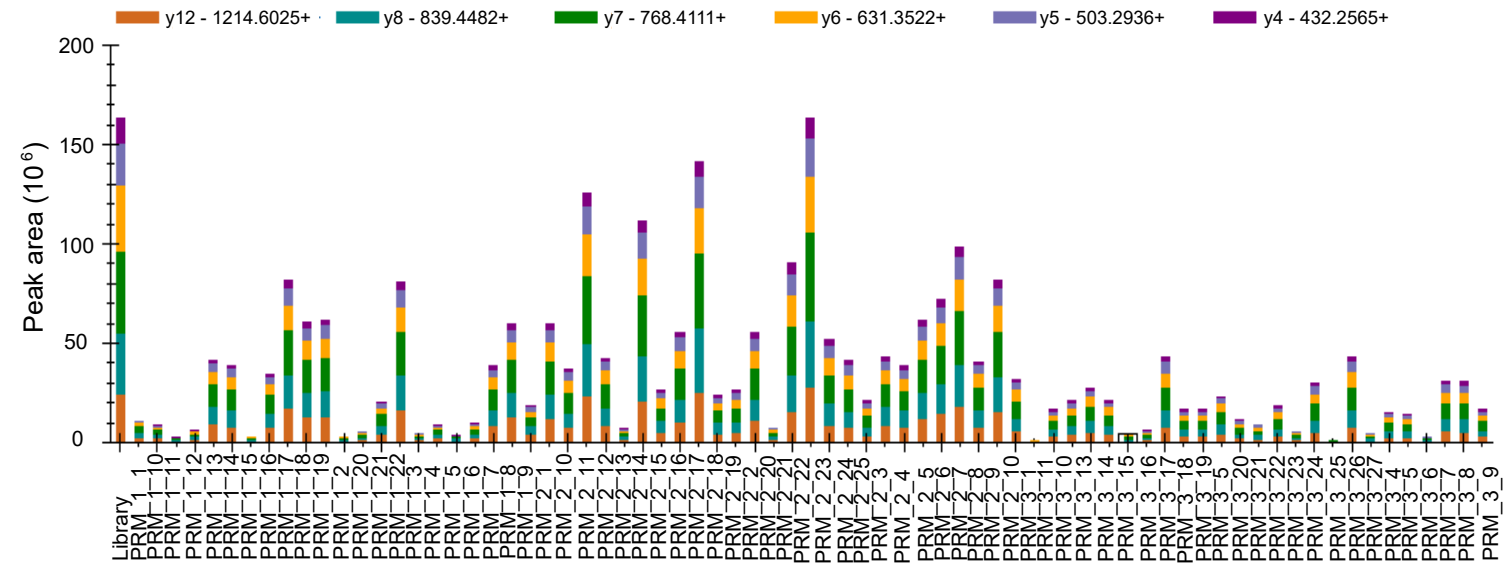

Supplement: Supplementary Figure S8 — Peak area for peptide LFGGNFAHQASVAR of TPP1 across 73 PRM runs in the PCa cohort. Relative abundance of TPP1 across 73 PRM runs in the PCa cohort was quantified using Skyline, with one representative example shown for LFGGNFAHQASVAR (m/z 737.88, 2+). A. XIC peak areas of the precursor ions obtained at MS1 level. B. XIC peak areas of fragment ions obtained at MS2 level. [file mmc8.pdf]

P02741, CRP (ESDTSYVSLK)

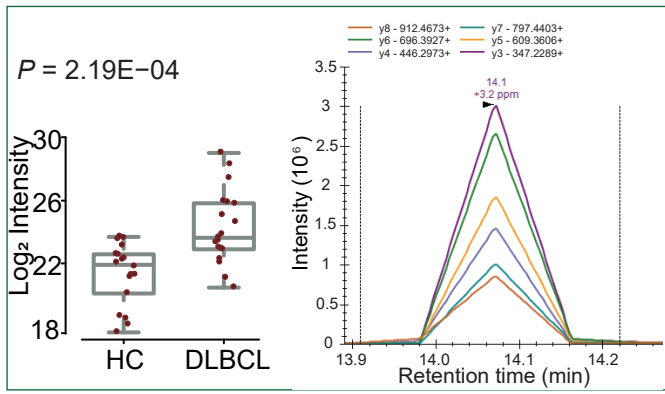

P02741, CRP (RQDNEILIFWSK)

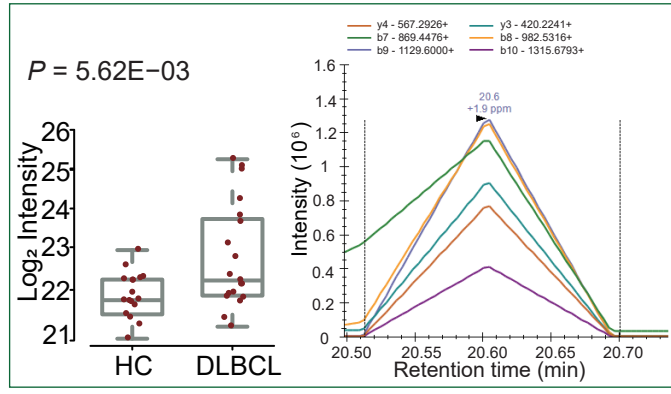

P0DJ18, SAA1 (FFGHGAEDSLADQAANEWGR)

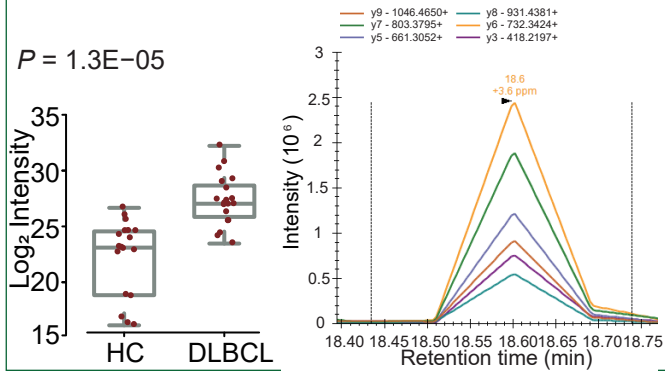

Supplement: Supplementary Figure S9 — PRM validation of CRP and SAA1 in the DLBCL cohort. Two best flying peptides were selected to quantify CRP (upper panels); one best flying peptide was selected for SAA1 (lower panels). Each box represents the information for one peptide. Box plots show the log2 MS2 intensity of the respective peptides across 37 plasma samples from the DLBCL cohort, including 19 DLBCL patients and 18 HC subjects (on the left), while XICs depict a representative peak group for each peptide from sample s8 (on the right). P values are computed using Student’s t test. [file mmc9.pdf]

**A XIC peak areas of precursor ions at MS1 level**

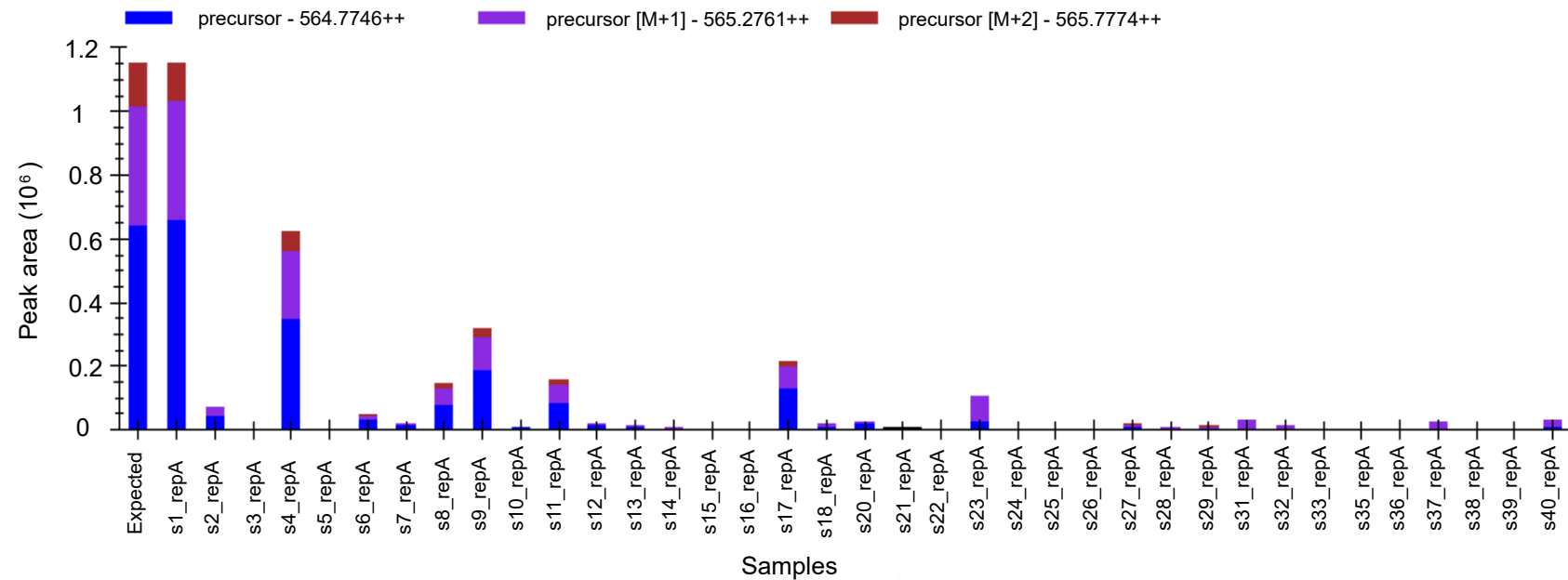

**B XIC peak areas of fragment ions at MS2 level**

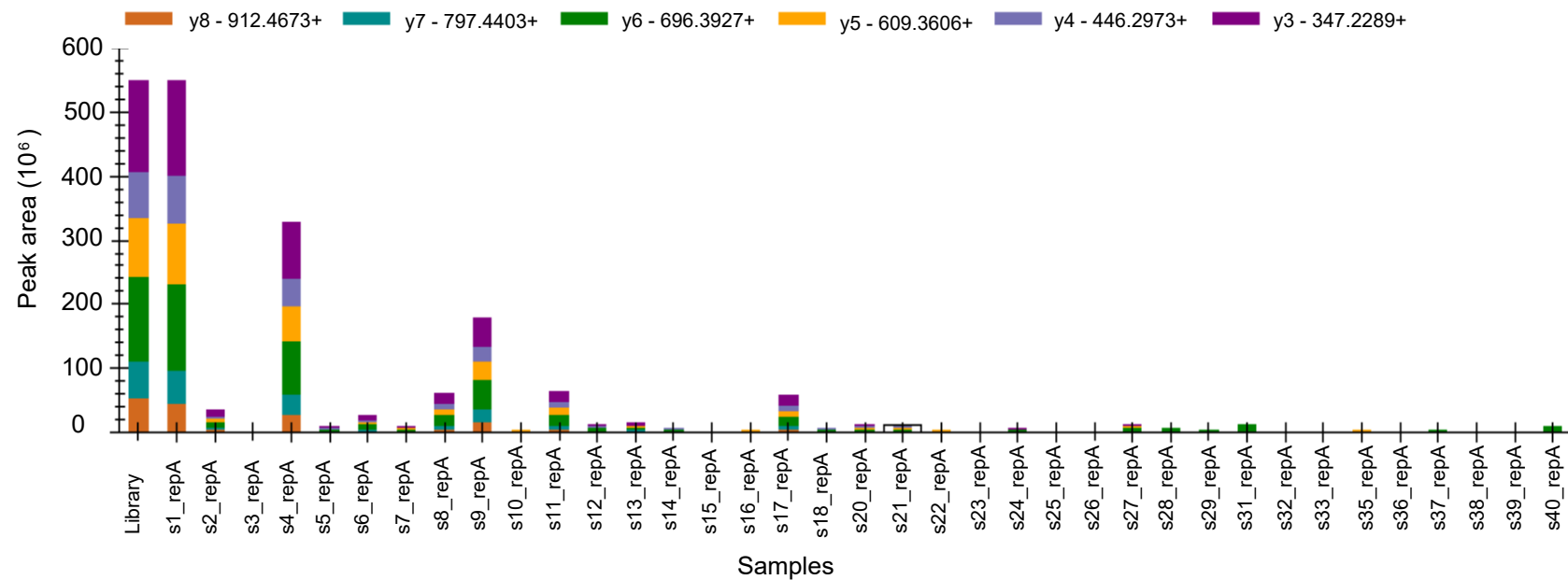

Supplement: Supplementary Figure S10 — Peak area for peptide ESDTSYVSLK of CRP across 37 plasma samples in the DLBCL cohort. The XIC peaks of the precursor ions at MS1 level (A) and fragment ions at MS2 level (B) for peptide ESDTSYVSLK (m/z 564.77, 2+) of CRP (P02741) were obtained from the DLBCL cohort, including 19 DLBCL patients and 18 HC subjects. [file mmc10.pdf]
