# Supplementary material for: DPHL: A DIA Pan-human Protein Mass Spectrometry Library for Robust Biomarker Discovery
Source: Genomics Proteomics Bioinformatics. 2020 Aug 12;18(2):104–19. doi: 10.1016/j.gpb.2019.11.008 (PMC7646093; doi:10.1016/j.gpb.2019.11.008)
Supplement: Supplementary Figure S2 — Accumulated datasets in DPHL. A. Number of transition groups (peptide precursors) in accumulated datasets in DPHL. The number of peptide precursors in DPHL increases cumulatively when adding datasets from various tissue types. B. Cumulative protein counts in DPHL. The number of proteins gets saturated as the number of raw data files increases. C. Cumulative peptide precursor and protein counts from pancreatic tissue samples based on 100 DDA files. Upper panel: number of peptide precursors; lower panel: number of proteins identified. D. Cumulative peptide precursor and protein counts from 179 plasma samples. Upper panel: number of peptide precursors; lower panel: number of proteins identified. [file mmc2.pdf]

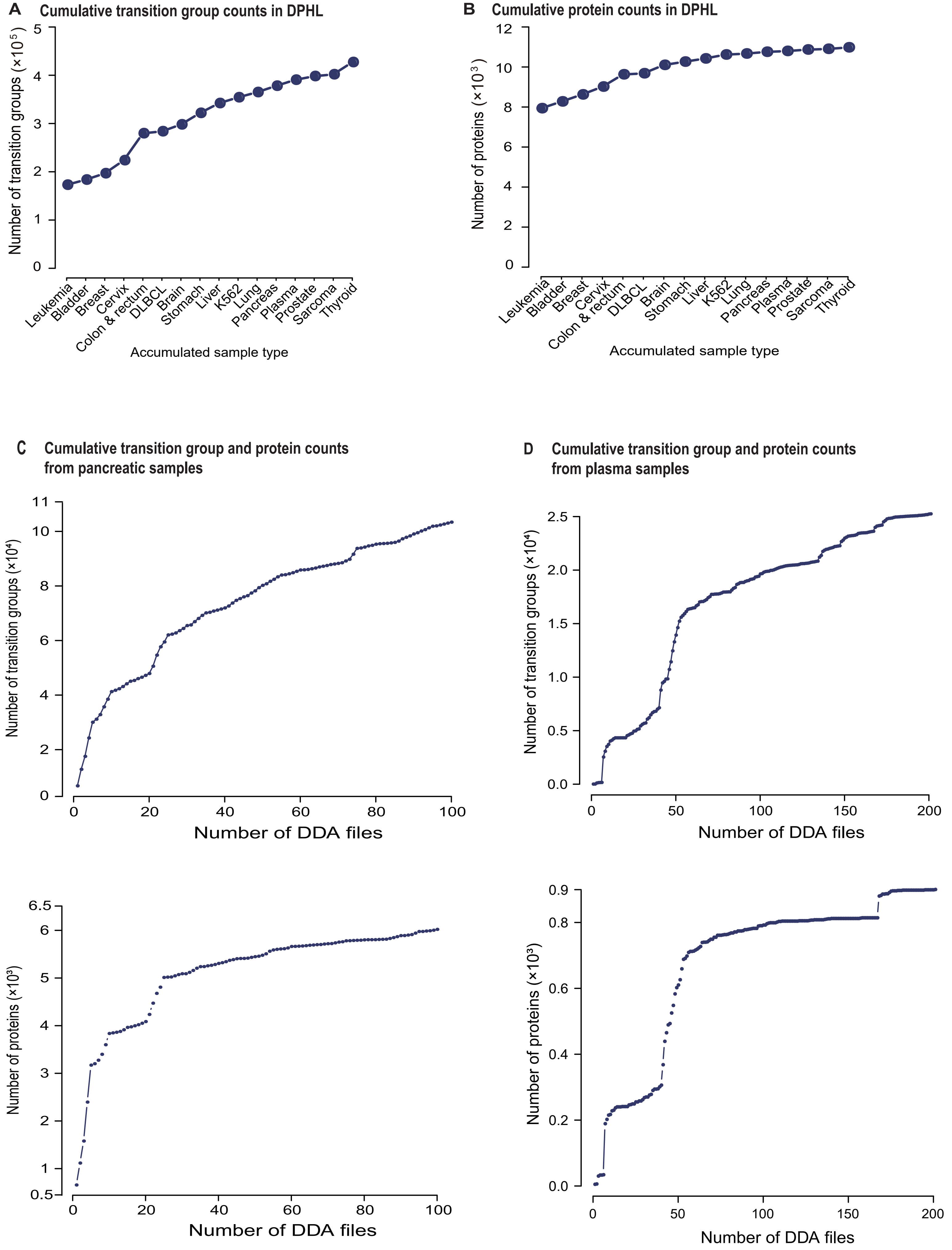
A
Cumulative transition group counts in DPHL
Number of transition groups (×10⁵)
Leukemia
Bladder
Breast
Cervix
Colon & rectum
DLBCL
Brain
Stomach
Liver
K562
Lung
Pancreas
Plasma
Prostate
Sarcoma
Thyroid
Accumulated sample type
B
Cumulative protein counts in DPHL
Number of proteins (×10³)
Accumulated sample type
C
Cumulative transition group and protein counts from pancreatic samples
Number of transition groups (×10⁴)
Number of DDA files
Number of proteins (×10³)
Number of DDA files
D
Cumulative transition group and protein counts from plasma samples
Number of transition groups (×10⁴)
Number of DDA files
Number of proteins (×10³)
Number of DDA files
