# Supplementary material for: DPHL: A DIA Pan-human Protein Mass Spectrometry Library for Robust Biomarker Discovery
Source: Genomics Proteomics Bioinformatics. 2020 Aug 12;18(2):104–19. doi: 10.1016/j.gpb.2019.11.008 (PMC7646093; doi:10.1016/j.gpb.2019.11.008)

A Number of proteins and kinases identified in DPHL

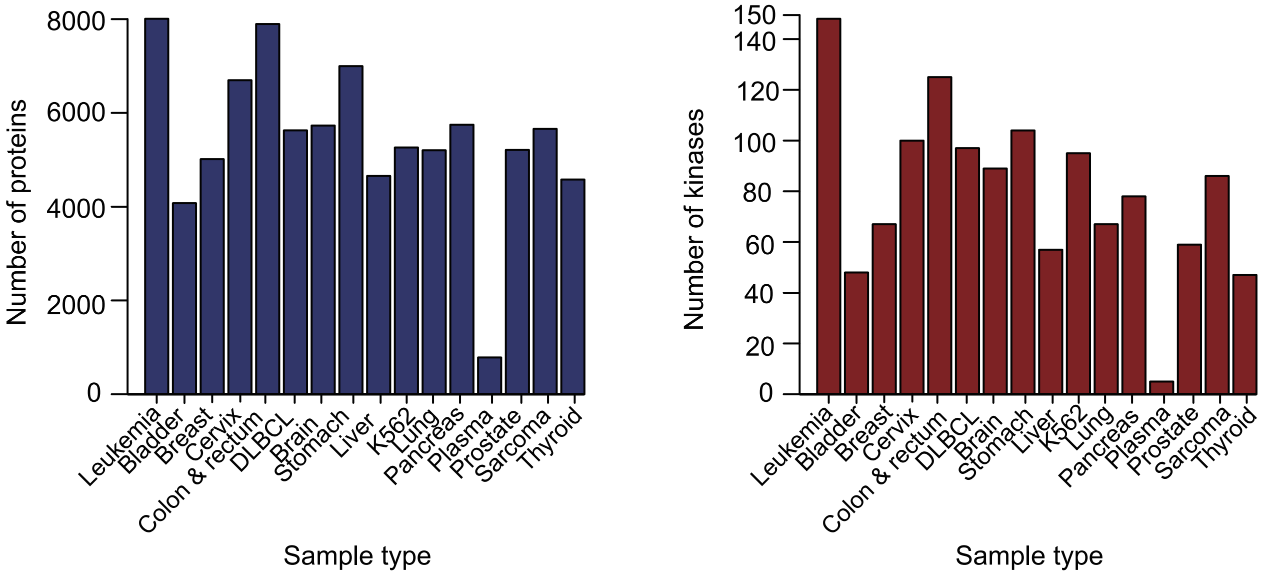

B Shared kinases for different tissue types

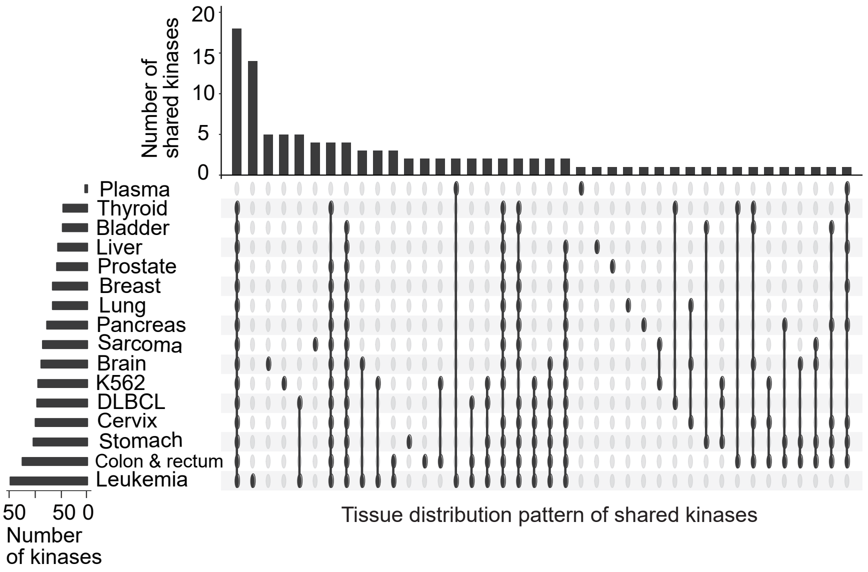

C Comparison of DPHL with Swiss-Prot and kinome

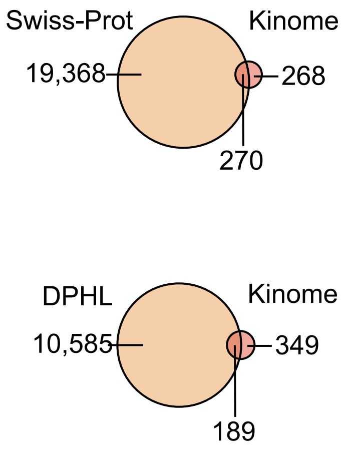

Supplement: Supplementary Figure S5 — Protein kinases identified in DPHL. A. Bar charts showing the number of proteins (left, blue) and the kinases (right, red) identified in the DPHL library. B. Overlap of kinases for different tissue types. C. Venn diagrams showing the comparison of DPHL with Swiss-Prot and the kinome. DPHL covers about 70% kinases in the Swiss-Prot database. [file mmc5.pdf]
