# Supplementary material for: DPHL: A DIA Pan-human Protein Mass Spectrometry Library for Robust Biomarker Discovery
Source: Genomics Proteomics Bioinformatics. 2020 Aug 12;18(2):104–19. doi: 10.1016/j.gpb.2019.11.008 (PMC7646093; doi:10.1016/j.gpb.2019.11.008)
Supplement: Supplementary File S1 — Comparison of DDA files acquired from the Guo lab and the Jimenez lab. [file mmc18.docx]

**File S18 Computational workflow for building DIA library**

**Part 1: Generate spectral library**

1. Obtain the DDA raw data for each tissue type.
2. Retrieve the fasta database. *e.g.* *swissprot_human_20180209_target_IRT_contaminant.fasta (we used this database downloaded from Swiss-Prot database on 9^th^ February, 2018 and added contaminants using pFind to build the DPHL)*
3. For every tissue type, follow steps 4 and 5.
4. Identify proteins and peptides using pFind (version 3.1.3).
   1. Parameters for pFind:
      1. Place of decimal: m/z = 3
      2. Uncheck mixture spectra
      3. Fixed modification = Carbamidomethyl [C]
      4. variable modification = Oxidation [M]
      5. enzyme = Trypsin_P KR P C
      6. fasta database as shown above
      7. default parameters for the other variables
   2. Obtain the *pFind.spectra* file after pFind is completed.
   3. Convert the raw data to mzXML data using ProteoWizard by running the following command:

*msconvert --mzXML --filter "peakPicking true [1,2] pathToRawData/*.raw*

1. Build the library.
   1. Copy pFind.spectra and all mzXML files to the DPHL docker
   2. Run the following command:

*build_library -i pFind.spactra -t iRT.txt*

**Part 2: Build up library with CiRT**

For MS data files without SiRT, users need to generate CiRT as follows.

1. Build the library as shown at the steps above using the samples with SiRT spike-in.
2. Run MaxQuant of representative samples, and obtain the result file *peptides.txt*.
3. Copy the *peptides.txt* and the csv/tsv format of the library built in Part 1.
4. Run the following command in the Docker.

*generate_CiRT dlbcl_peptides.txt dlbcl_library.csv dlbcl*

NOTE: “dlbcl” in this command can be replaced by other tissue type of interest.

1. Generate the CiRT library *dlbcl_CiRT.txt* (tsv).
2. Replace the iRT.txt in the step 5 in Part 1 with this *dlbcl_CiRT.txt* file, and run the step 5 in Part 1.

**Part 3: Build the final library**

When all the sub-libraries for each sample type are generated, build the consensus library by running the following command:

*buildFinalLibrary.py -i <inputfiles splibs, blank separated and all in one quotes > -w <DIA windows file name.default "/swath/mnt/windows_QE_HF.txt">*

*-o <outputfile name,Optional,default Finallibrary> -c <number of threads, default 4> -h <help>*

For example:

*buildFinalLibrary.py -i dlbcl.splib stomach.splib -w /swath/mnt/windows_QE_HF.txt*

*-o final_consensus*

**Part 4: Add new DDA files to library**

1. Follow the instructions in Part 1.
2. Build the final library as shown in Part 2.

For example:

*buildFinalLibrary.py -i* *newadd_consensus.splib dphlv1.splib -o final_new_library*

Note: *newadd_consensus.splib was the* *interim output file in part 4 step 1 and (*pepidx and spidx suffix files should be in the same folder with splib file*), dphlv1.splib was the interim file of DPHL library, provided in supplementary files.*

**Part 5: Build library for advanced users**

NOTE: In order to simplify the workflow to build library, we embed many optimized parameters in the pipeline as default and leave few ones for users to adjust. If users want to adjust or check all parameters, please follow the instructions below:

1. Use “which” command to get the path of “build_library”: “which build_library”.
2. Then use “vi” to show or adjust advanced parameters in the script: “vi /path/to/build_library”.
3. Modify the parameters as wish.
