# Supplementary material for: DPHL: A DIA Pan-human Protein Mass Spectrometry Library for Robust Biomarker Discovery
Source: Genomics Proteomics Bioinformatics. 2020 Aug 12;18(2):104–19. doi: 10.1016/j.gpb.2019.11.008 (PMC7646093; doi:10.1016/j.gpb.2019.11.008)

**File S19 Practical guide using test data as an example**

1. **Install docker and import images**
2. **Install Docker**

Whatever the operation system you are using, the first thing is to install Docker. Docker can be installed from the software repository.

**For Ubuntu,** use the following shell command:

apt-get install docker.io

**For RedHat/CentOS**:

yum install docker

systemctl enable docker

systemctl start docker

**For Windows and Mac:**

Docker Desktop is the easiest way to run Docker Engine, Docker Swarm, and Kubernetes on Mac and Windows.

Go to <https://www.docker.com/products/docker-desktop> and download the right version for your system. Then double-click the downloaded file and install it.

1. **Import Docker image and start the container**

Use the following command to import Docker container, you can download DPHL_container.tar from the site given in the main text.

Login in Linux server

Then import Docker container

cat DPHL_container.tar | docker import - dphl:v01

Check import

docker images

Start and login in the docker dphl:v01

docker run -i –t dphl:v01 /bin/bash

1. **Check and prepare the test data**

List the test file

**ls**

Decompression

tar -zxvf testData.tar.gz


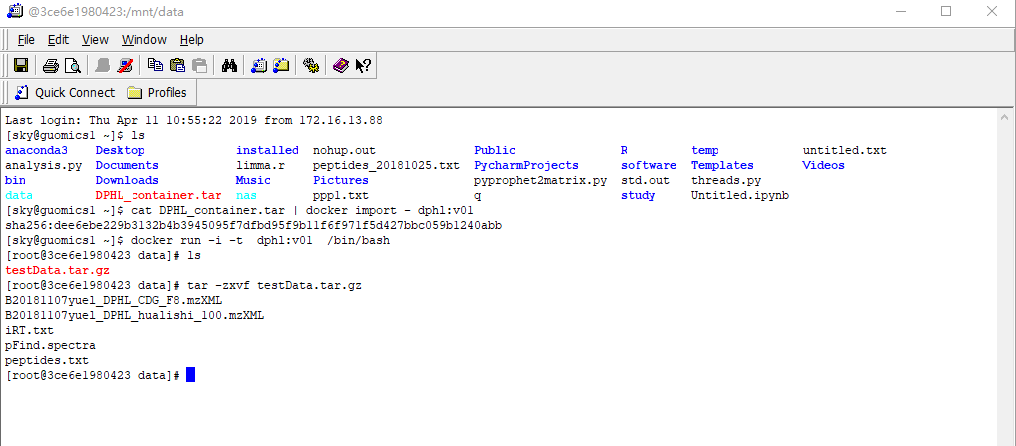


1. **Building library**

Now, the current directory has two test mzXML files: B20181107yuel_DPHL_CDG_F8.mzXML, B20181107yuel_DPHL_hualishi_100.mzXML; the peptides.txt, which is the MaxQuant result file of these two mzXML files; the pFind.spectra, which is the pFind result file of these two mzXML files; and an iRT.txt file.

1. Building library using SiRT

build_library -i pFind.spectra –t iRT.txt -o testLib


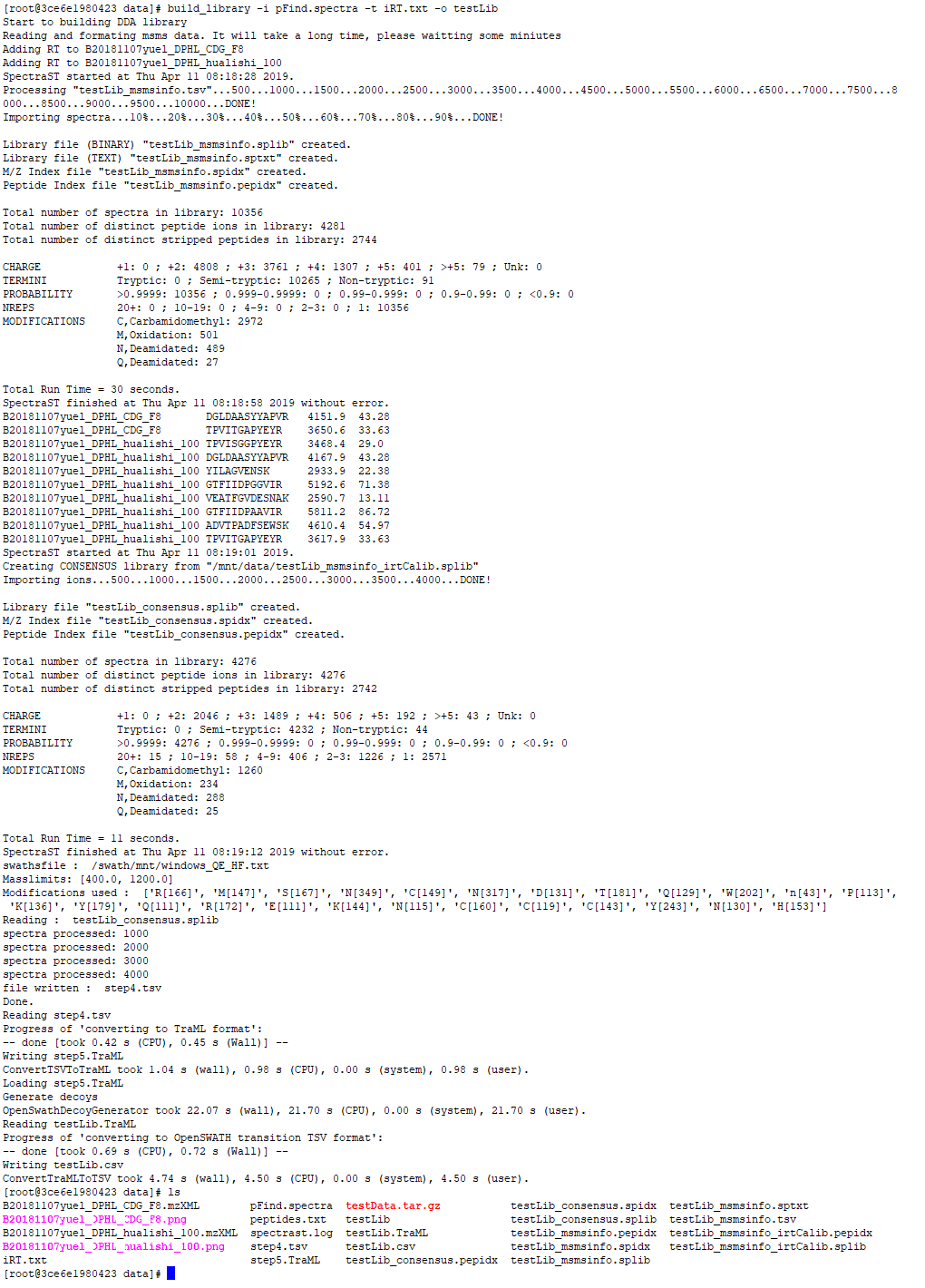


1. Generating CiRT

Here we suppose these two mzXML files don’t contain SiRT and a testLib was built by the other files with iRT spike-in.

generate_CiRT peptides.txt testLib_pFind.csv CiRT


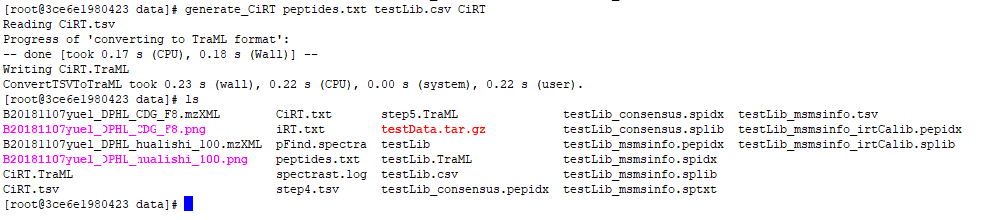


1. Building library with CiRT

build_library -i pFind.spectra -t CiRT.txt -o testLib2


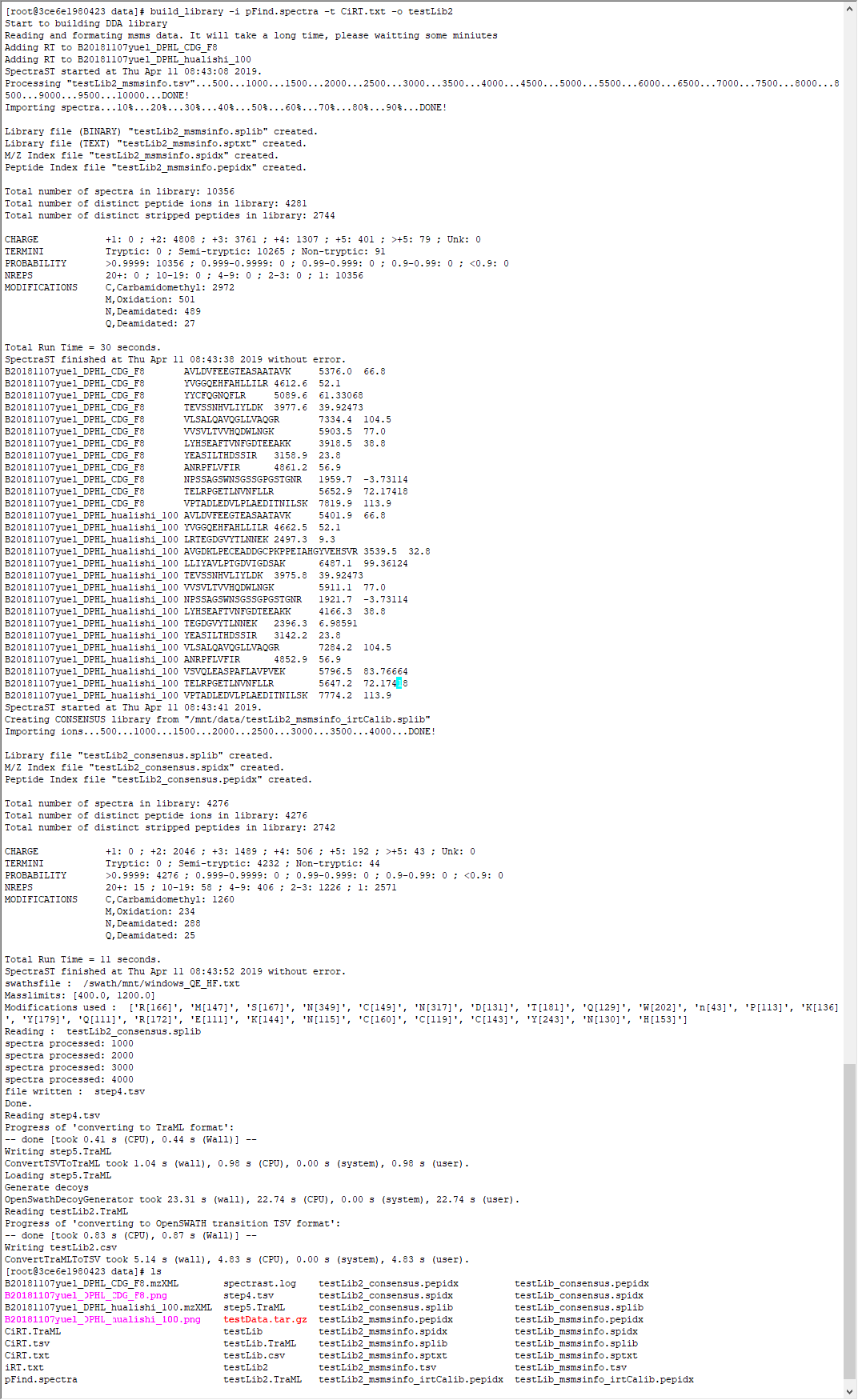

Supplement: Supplementary File S2 — TraML format of bladder CiRT. [file mmc19.docx]
